# Multi‐Omic Analysis Reveals Astrocytic Annexin‐A2 as Critical for Network‐Level Circadian Timekeeping in the Suprachiasmatic Nucleus

**DOI:** 10.1002/glia.70018

**Published:** 2025-04-02

**Authors:** Andrew P. Patton, Toke P. Krogager, Elizabeth S. Maywood, Nicola J. Smyllie, Emma L. Morris, Mark Skehel, Michael H. Hastings

**Affiliations:** ^1^ Division of Neurobiology Medical Research Council Laboratory of Molecular Biology Cambridge UK; ^2^ Department for Neural Systems and Coding Max Planck Institute for Brain Research Frankfurt am Main Germany; ^3^ Medical Research Council Laboratory of Molecular Biology Cambridge UK; ^4^ Proteomics Science Technology Platform The Francis Crick Institute London UK

**Keywords:** Anxa2, astrocytes, circadian, neurons, proteomics, S100a10, SCN

## Abstract

The mammalian suprachiasmatic nucleus (SCN) orchestrates daily (circadian) rhythms of physiology and behavior by broadcasting timing cues generated autonomously by its mutually reinforcing network of ~10,000 neurons and ~3000 astrocytes. Although astrocytic control of extracellular glutamate and GABA has been implicated in driving circadian oscillations in SCN gene expression and neuronal activity, the full scale of the network‐level signaling mechanisms is unknown. To understand better how this astrocyte‐neuron network operates, we adopted a multi‐omics approach, first using SILAC‐based mass spectrometry to generate an SCN proteome where ~7% of identified proteins were circadian. This circadian proteome was analyzed bioinformatically alongside existing single‐cell RNAseq transcriptomic data to identify the cell‐types and processes to which they contribute. This highlighted “S100 protein binding,” tracked to astrocytes, and revealed annexin‐A2 (Anxa2) as an astrocyte‐enriched circadian protein for further investigation. We show that Anxa2 and its partner S100a10 are co‐expressed and enriched in SCN astrocytes. We also show that pharmacological disruption of their association acutely and reversibly dysregulated the circadian cycle of astrocytic calcium levels and progressively compromised SCN neuronal oscillations. Anxa2 and S100a10 interaction therefore constitutes an astrocytic cellular signaling axis that regulates circadian neuronal excitability and ultimately SCN network coherence necessary for circadian timekeeping.

## Main Points

- Multi‐omics reveal Annexin‐A2 (A2) and S100a10 (A10) as SCN astrocyte enriched proteins.
- Disrupting A2‐A10 complex elevates astrocyte Ca^2+^ and compromises SCN timekeeping.
- Astrocytic A2‐A10 is a critical control point for SCN network rhythms.

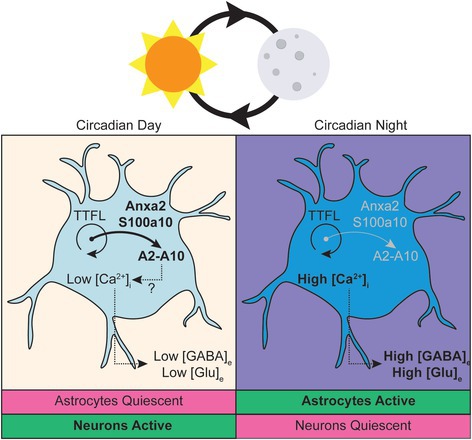

## 1 Introduction

The hypothalamic suprachiasmatic nucleus (SCN) is the principal mammalian circadian pacemaker, conveying temporal cues across the body and thus coordinating physiological and behavioral rhythms that adapt individuals to the demands of daily environmental cycles (Reppert and Weaver 2002). Each of the paired nuclei contains ~10,000 neurons and 3000 glia, principally astrocytes (Guldner 1983). Together, they form a powerful network that can maintain an internal representation of time in the absence of environmental input, even when isolated as an explant (Hastings et al. 2018). The SCN achieves this remarkable feat due to the interactions between cell‐autonomous oscillators of the SCN and the neuronal and astrocytic networks (Astiz et al. 2021; Smyllie et al. 2025).

The cell‐autonomous oscillator of SCN (and all nucleated) cells pivots around a core transcriptional‐translational feedback loop (TTFL), whereby transcription of negative regulators *Period* (*Per*) and *Cryptochrome* (*Cry*) is activated by the heterodimerized transcription factors CLOCK and BMAL1. The transactivation allows Per and Cry proteins to accumulate across the circadian day, entering the nucleus and repressing CLOCK/BMAL1‐mediated transcription. This terminates Per and Cry production, their levels falling over the circadian night to the point where CLOCK/BMAL1 repression is released approximately 24 h later (Cox and Takahashi 2019). Ultimately, cyclical activation and repression of these transcription factors organizes daily programmes of “clock‐controlled” genes that coordinate circadian neural activity patterns (Hastings et al. 2018).

In the SCN, the TTFL is supported by intercellular signals acting through Ca^2+^/cAMP response elements (CREs) in the *Per* gene promoters, boosting the amplitude of the cell‐autonomous TTFL. Thus, clock outputs, including electrical activity and neuropeptidergic signaling, become inputs, thereby increasing robustness (Patton, Hastings, et al. 2023). Robustness is further conferred by network‐level interplay between neurons and astrocytes in which astrocytes facilitate daytime neuronal activity and suppress night‐time neuronal activity by modulating GABAergic signaling. This modulation has been shown to occur through daytime uptake of GABA (Patton, Morris, et al. 2023) and is reported to involve astrocytic GABA synthesis (Ness et al. 2024). The full scale of cellular mechanisms and signaling pathways involved in astrocyte‐neuronal coordination is, however, unknown: which rhythmically expressed SCN genes and proteins support network oscillation, and in which cell‐types are they situated?

To start addressing this in an unbiased manner, we adopted a “multi‐omics” approach in which we identified circadian‐regulated proteins in SCN organotypic slices using quantitative Stable Isotope Labeled Amino acid in Cell culture (SILAC)‐based mass spectrometry (MS) (Chiang et al. 2014). Having identified circadian proteins, we cross‐referenced them to a pre‐existing single‐cell transcriptomic dataset from a comparable SCN preparation (Morris et al. 2021) to assign proteins and molecular functions of interest to specific SCN cell‐types. This highlighted the astrocyte‐enriched protein annexin‐A2 (Anxa2) and its associate S100a10. In situ hybridization of organotypic SCN confirmed their co‐expression in astrocytes, indicating their interaction as a clock‐regulated signaling node. Using specific and reversible pharmacological disruption of Anxa2/S100a10 interaction, we demonstrated its functional relevance to SCN network‐level timekeeping: in its absence, circadian rhythmicity of astrocytic calcium levels was acutely disrupted. Furthermore, over several days, the circadian activity of the network‐level TTFL was initially phase‐shifted and then progressively suspended. These results extend our knowledge of how astrocytes and neurons interact to control the SCN clockwork, showing that Anxa2 and S100a10 interactions constitute an astrocytic cellular signaling axis that regulates circadian astrocytic activity, neuronal excitability, and SCN network coherence and is necessary for circadian timekeeping.

## 2 Materials and Methods

### 2.1 Animals

All mouse work was carried out in accordance with the UK Animals (Scientific Procedures) Act of 1986, with local ethical approval from the Animal Welfare and Ethical Review Body (AWERB) of the Medical Research Council (MRC) Laboratory of Molecular Biology (LMB). Mice were maintained and bred at the LMB Ares facility and were maintained on a 12:12 light:dark schedule with food and water available ad libitum. Period2::Luciferase (Per2::Luc) mice (Yoo et al. 2004) were a gift from Dr. J.S. Takahashi (University of Texas Southwestern Medical Center, Dallas, TX) back‐crossed in‐house onto C57BL/6J mice (Jackson Laboratories), which were also used as wild‐types.

### 2.2 Organotypic Slice Preparation

Mice of postnatal day 10 (P10) to P12 and of either sex were killed, and SCN explants were prepared as previously described (Hastings et al. 2005; Patton et al. 2020). The brain was removed and transferred to ice‐cold Gey's Buffered Saline Solution (GBSS) supplemented with 5 mg/mL glucose, 3 mM MgCl_2_, 0.05 mM DL‐AP5, and 100 nM (+)‐MK‐801. It was then trimmed into a block containing the hypothalamus before 300 μm thick coronal slices were made on a tissue chopper (McIlwain, UK). The suprachiasmatic nucleus (SCN) was dissected from the coronal hypothalamic slices before being cultured as an explant via the interface method on tissue culture inserts (Millipore). The slices were rested in culture medium (CM) consisting of 50% Eagle's Basal Medium, 25% Earle's Balanced Salt Solution, and 25% horse serum supplemented with 5 mg/mL glucose, 2 mM Glutamax (Gibco), penicillin/streptomycin (Gibco) and, additionally, 3 mM MgCl_2_, 0.05 mM DL‐AP5, and 100 nM (+)‐MK‐801 for a few hours before being transferred to HEPES‐buffered Dulbecco's Modified Essential Medium‐based recording medium (AM) supplemented with Glutamax, penicillin/streptomycin, fetal calf serum (FCS) and B‐27 for at least 1 week before use.

### 2.3 Stable Isotope Labeling by Amino Acids in Cell Culture (SILAC) Labeling

**FIGURE 1:**
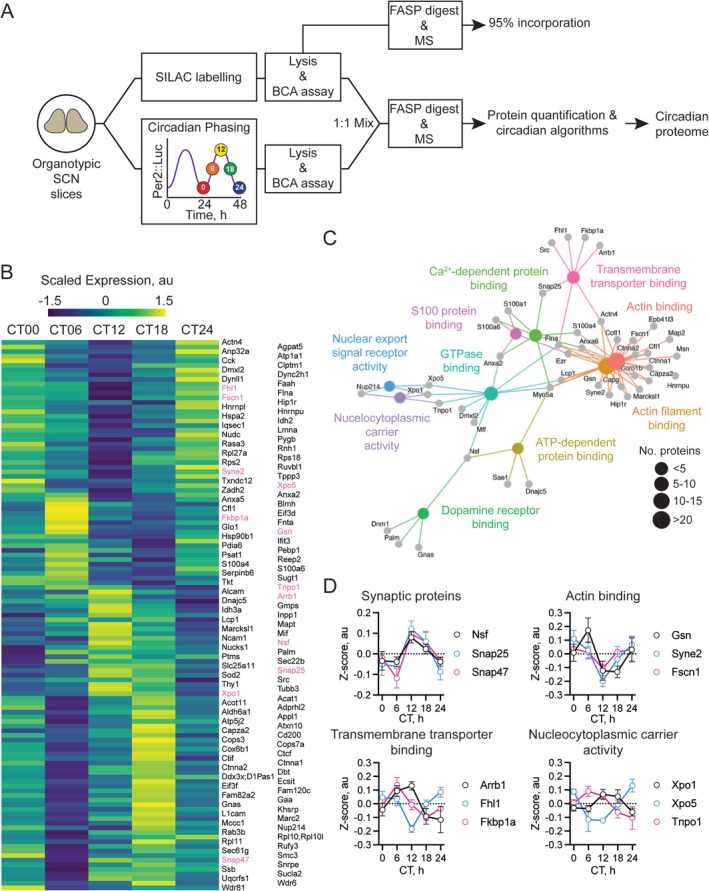
Circadian proteomic analysis of free‐running SCN explants. (A) Schematic depicting the pipeline for the generation of a SCN circadian proteome. Wild‐type C57BL/6J and Per2::Luc pups were used to produce organotypic SCN explants. Upper path: Wild‐type explants were cultured in SILAC medium to produce an endogenous heavy spike‐in reference sample resulting in ~95% incorporation. Lower path: Per2::Luc slices were harvested in 2% SDS at CT0, 6, 12, 18, and 24 at times extrapolated from their bioluminescence rhythms. The samples from slices at different CTs were mixed 1:1 with the heavy‐labeled spike‐in and digested by the FASP method before individual SILAC ratios were quantified by LC–MS/MS. Proteins were then quantified, and the circadian proteome was defined by the application of the circadian algorithms RAIN and JTK‐cycle. (B) Heatmap showing scaled expression levels of all 153 proteins identified as circadian in our sample, k‐means clustered to show distribution across the phases of collection. Protein names are shown on the right, with odd‐numbered rows shown in the left‐hand column and even‐numbered rows in the right‐hand column. Protein profiles shown in more detail (D) are colored pink. (C) A category network plot for the circadian proteome showing the top 10 most significant MF‐GO terms and the proteins in the dataset that contribute to them, arranged as an interaction network. (D) Example circadian expression profiles for proteins with similar biological functions defined in previous findings (Deery et al. 2009) or by the MF‐GO terms in (C). Exemplars are shown for synaptic proteins (upper left), actin binding (upper right), transmembrane transporter binding (lower left) and nucleocytoplasmic carrier activity (lower right).

To heavy‐label SCN slices, a specially formulated SILAC medium based on our CM was used, with the substitution of custom DMEM lacking arginine and lysine (AthenaES), 10% heat‐inactivated horse serum dialyzed (10 kDa, Spectrum Lab) against 50 mM NH_3_HCO_3_ (Invitrogen), and 0.398 μM Arg + 10 and 0.798 μM Lys + 6 (Cambridge Isotope Laboratories). Wild‐type slices were maintained in this SILAC CM for 8 weeks to create a time‐free SILAC SCN “spike‐in” proteome. The labeling efficiency was measured to ≈95%, based on the number of heavy peptides divided by the number of light peptides (Chen et al. 2015) (LFQ vs. peptide count) (Figure 1A).

### 2.4 Bioluminescence Recording

During recording, slices were maintained in AM supplemented with 100 μM luciferin in dishes sealed with glass coverslips (Patton et al. 2020). Aggregate bioluminescence was monitored in real‐time in customized light‐tight incubators with individual photon‐multiplier tubes (PMTs; catalogue #H9319‐11 photon counting head, Hamamatsu). Data collected were binned into 6‐min epochs for export and analysis.

### 2.5 Harvesting of SCN Slices for Proteomics

At least 1 week after preparation, Per2::Luc slices were transferred to PMTs to record their free‐running bioluminescence. After several circadian cycles, the circadian phase was determined via the Per2::Luc bioluminescence signal, and slices were harvested serially at CT0, 6, 12, 18, and 24 (i.e., CT0 repeat). Thus, although the CT time stamp was absolute, it would have been meaningless to refer to “cycle 1” and “cycle 2” as all slice samples were free‐running independently without common registration to external time. This structure necessitated that our analysis was limited to a one‐cycle design, which therefore informed how we should apply circadian algorithms to analyze our data.

### 2.6 Lysis and Digest

Slices were washed with 0.01 M Phosphate Buffered Saline (PBS) and then mechanically aspirated before lysis in 2% SDS in Tris‐buffered saline (TBS) (pH 7.4, 5 μL/slice). Seven SCN slices were pooled per triplicated sample and boiled for 5 min before sonication in a water bath for 30 s. After centrifugation, the protein concentration of the supernatant was determined by BCA assay (Pierce). The unlabeled circadian time‐point samples were mixed 1:1 with the SILAC‐labeled SCN lysate; subsequently, 50 μg of the protein mix was digested by the FASP method (Wisniewski 2017) with 1 μg trypsin (Sigma).

### 2.7 Mass Spectrometry

The resulting peptide mixtures were acidified by the addition of formic acid (final concentration 2% v/v). The digests were analyzed in triplicate (3 μg initial protein/injection) by nano‐scale capillary LC–MS/MS using an Ultimate U3000 HPLC (ThermoScientific Dionex) to deliver a flow of approximately 300 nL/min. A C18 Acclaim PepMap100 5 μm, 100 μm × 20 mm nanoViper (ThermoScientific Dionex) trapped the peptides prior to separation on a C18 PepMap100 3 μm, 75 μm × 250 mm nanoViper (ThermoScientific Dionex). Peptides were eluted with a 120 min gradient of acetonitrile (2%–60%). The analytical column outlet was directly interfaced via a nano‐flow electrospray ionization source, with a hybrid quadrupole orbitrap mass spectrometer (Q‐Exactive Plus Orbitrap, ThermoScientific).

Data‐dependent analysis was carried out using a resolution of 30,000 for the full MS spectrum, followed by 10 MS/MS spectra. MS spectra were collected over an m/z range of 300–2000. MS/MS scans were collected using a threshold energy of 27 for higher energy collisional dissociation (HCD). MS raw files were processed by MaxQuant (Tyanova et al. 2016) version 1.3.0.5 and searched with Andromeda (Cox et al. 2011) against a reviewed mouse proteome retrieved from Uniprot (Oct 2014, 16,650 entries). Searches were done with an MS/MS tolerance of 20 ppm and 1% FDR at both the peptide and protein levels. Carabamidomethylation of cysteine was set as a fixed modification, and oxidation of methionine and protein N‐terminal acetylation were chosen as variable modifications. Two missed cleavages were allowed. One quantified peptide per protein group was allowed since further statistical analysis on the biological replicates was applied. Common contaminants were removed and further filtered and processed with R statistical software (R). As a criterion for quantification, at least two‐SILAC ratios per time‐point were required and SILAC ratios were log2 transformed.

### 2.8 Circadian Analysis of Proteomic Data

Quantified proteins were analyzed for circadian oscillation using two different and widely used circadian algorithms: RAIN (version 1.34.0) (Thaben and Westermark 2014) and JTK‐Cycle (version 3.1) (Hughes et al. 2010). Both RAIN and JTK‐Cycle were implemented in R running in the RStudio environment. Due to the nature of our data (low‐frequency sampling over 1 cycle with an independently repeated CT24/0), we used a stringent, conservative criterion such that a protein in our dataset could only be considered circadian if it was significant by both RAIN (*p* < 0.05) and JTK‐Cycle (*p* < 0.05) (Figure S1), and the overlap was determined using the VennDiagram package (version 1.7.3) (Chen and Boutros 2011).

The expression data heatmap was produced in R using the pheatmap package (version 1.0.12) (Kolde 2019), implementing k‐means clustering to cluster the data into the four expected phases that could reasonably be expected from the experimental structure (peaking at CT0/24, 6, 12 or 18). Data represented in the heatmap are rescaled by protein to reveal the dynamics without consideration of different oscillation amplitudes.

### 2.9 Analysis of Single‐Cell RNA‐Seq (scRNA‐Seq) Data

Publicly available data from a previously published scRNA‐seq dataset produced from free‐running SCN explants (Morris et al. 2021) were accessed from the NCBI Gene Expression Omnibus with the accession number: GSE167927. These data were re‐analyzed in R using the Seurat package (version 5.1.0) (Hao et al. 2021, 2024) to generate a cell‐type‐specific transcriptome. Data were first filtered to remove cells containing high levels of mitochondrial transcripts before data were log‐normalized and scaled using the *NormalizeData* and *ScaleData* functions. The data were then clustered using *FindNeighbors*(dim = 40) and *FindClusters*(resolution = 1.2, method = “igraph,” algorithm = 3) before clusters were assigned identities based on their expression of cell‐specific marker genes to allow further analysis of cell‐type specific transcriptomes.

Cell‐type enrichment of Anxa2 and potential interaction partners were examined by first identifying Anxa2 interaction partners using the BioGRID database of curated protein–protein interactions (www.thebiogrid.org, BioGRID version 4.4.235; Oughtred et al. 2021; Stark et al. 2006). This analysis was filtered for interactions identified in *Mus musculus* , and a comprehensive list of 40 potential interactors (including Anxa2) was queried against the cell‐type clustered single‐cell transcriptomic dataset using the *FetchData* function in the Seurat package and pulling the expression data from the counts data layer along with the cell identities. These were then summed across cell‐types and min‐max normalized across genes using the caret package (version 6.0‐94) (Kuhn 2008). Finally, a heatmap was generated using the *pheatmap* function. A differential expression test for day‐night expression of astrocyte transcripts was performed using the built‐in Seurat *FindMarkers* function, using the Wilcox test, subsetting the Seurat object to include only astrocytes and grouping the data by time of day of harvesting, which was added to the metadata. Volcano plots were generated using the EnhancedVolcano package (version 1.18.0) (Blighe et al. 2024).

### 2.10 Bioinformatic Analysis

GO‐term analysis was carried out in R using the clusterProfiler package (version 4.8.3) (Wu et al. 2021). To determine the cluster‐specific GO‐terms from the scRNA‐seq cell clusters, first, a differential expression test was performed for specific clusters using the Seurat function *FindAllMarkers* with the only.pos = TRUE option to find transcripts whose expression (presence) defines those cells. These results were then filtered to include only significant (adjusted *p* < 0.05) transcripts and ordered by significance. From this list, the top 250 genes in each cluster were passed to the GO‐term analysis function. These cell‐specific lists were analyzed using the *compareCluster* function from clusterProfiler, using the enrichGO option to specify the molecular function (MF) ontology and *Mus musculus* organism database (org,Mm.eg.db, version 3.17.0; Carlson 2023). Even though 11 cell‐type clusters were identified in our transcriptomic dataset, due to overlap in the top two GO‐terms reported for closely related cell‐clusters, 19 final GO terms were identified, not the expected 22.

To determine the MF GO‐terms for the circadian proteome data, astrocyte transcriptome, and intersection between the two, gene lists were passed to the *enrichGO* function, specifying the *Mus musculus* organism database and MF ontology as above. The top 10 most significant GO‐terms were then passed to the *cnetplot* function from the enrichplot package in R to plot category network plots enabling the visualization of contributing genes/proteins.

### 2.11 Hybridization Chain Reaction (HCR) Fluorescent In Situ Hybridization (FISH)

Probe sets against *S100a10* (B1 adaptor, designed from NM_009112.2, 12 probes), *Anxa2* (B2 adaptor, designed from NM_007585.3, 23 probes) and *Sox9* (B3 adaptor, designed from NM_011448.4, 20 probes) were designed and manufactured by Molecular Instruments. SCN slices were excised from the culture insert and fixed in 4% PFA for 1 h before being washed three times for 15 min each in PBS‐T (0.01 M PBS supplemented with 0.1% Tween‐20). Slices were then dehydrated in a series of graded methanol washes: 100%:0%, 75%:25%, 50%:50%, 25%:75%, and 0%:100% PBS‐T:methanol, before rehydration through the same series of washes in reverse: 0%:100%, 25%:75%, 50%:50%, 75%:25%, and 100%:0% PBS‐T:methanol. These graded washes were incubated for 15 min on ice per step. Slices were then incubated in pre‐warmed Probe Hybridization buffer (Molecular Instruments) supplemented with 1:20 RNAse Inhibitor, Murine (New England Biolabs) for 1 h at 37°C before being incubated with 40 nM S100a10‐B1, 40 nM Anxa2‐B2, and 8 nM Sox9‐B3 probe sets in Probe Hybridization Buffer supplemented with 1:20 RNAse Inhibitor, Murine at 37°C for 24 h.

Primary probes were removed from slices by three washes in pre‐warmed Probe Wash Buffer (PWB, Molecular Instruments) at 37°C for 15 min each before additional graded 5× SSC with 0.1% Tween‐20 (SSC‐T) and PWB, wash steps at 37°C for 15 min each: 0%:100%, 25%:75%, 50%:50%, 75%:25%, 100%:0% SSC‐T:PWB. Finally, slices were run through a further three SSC‐T washes for 15 min at each step at room temperature before incubation in Amplification Buffer (Molecular Instruments) supplemented with 1:20 RNAse Inhibitor, Murine for 1 h at room temperature. Half an hour before the end of the incubation with Amplification Buffer, fluorescently labeled hairpins were snap‐cooled by heating individual hairpins to 95°C for 90 s in a thermocycler, before immediately transferring tubes to a dark drawer to cool over 30 min. Once cooled, 60 nM B1‐Alexa‐647 (h1/h2), B2‐Alexa‐594 (h1/h2), and B3‐Alexa‐488 (h1/h2) amplifiers (Molecular Instruments) were added to Amplification Buffer supplemented with 1:20 RNase Inhibitor, Murine, and slices were incubated for an additional 24 h at room temperature.

Secondary probes were removed by eight sequential SSC‐T washes for 15 min each. Slices were incubated in SSC‐T supplemented with 1 μg/mL DAPI (Tocris, UK) for 1 h before three more SSC‐T washes for 5 min each. Slices were then briefly rinsed in ddH_2_O before mounting onto glass slides with Vectashield Vibrance Antifade Mounting Medium (H‐1700, Vector Laboratories).

### 2.12 Adeno‐Associated Viral Vectors (AAVs) and Transduction

GFAP.jGCaMP7b was a gift from Thomas Oertner (Lohr et al. 2021) (Addgene plasmid # 171118; http://n2t.net/addgene:171118; RRID: Addgene_171118) packaged into AAV serotype 5 particles by VectorBuilder (> 1 × 10^13^ GC/mL). Syn.NES‐jRCaMP1a was a gift from Douglas Kim & the GENIE project (Addgene viral prep # 100848‐AAV1; http://n2t.net/addgene:100848; RRID: Addgene_100848) (Dana et al. 2016).

At least 1 week post‐culturing, organotypic SCN slices were transduced with AAV particles by dropping 1 μL AAV directly on top of the SCN slice. Slices were placed in an incubator and super‐transduced with further AAVs in the same manner at an interval of 2–3 days to express different reporters. AAVs were washed out of the slice by complete medium change.

### 2.13 Confocal Microscopy of Fluorescent Proteins

AAV‐expressed fluorescent reporters were imaged in slices without staining. Slices were fixed in 4% PFA diluted in 0.01 M phosphate buffer for 1 h before three washes in PBS‐T for 15 min each wash. Slices were incubated in PBS‐T supplemented with 1 μg/mL DAPI for an hour before being washed three times in PBS‐T for 5 min each wash and finally rinsed in ddH_2_O. Slices were mounted on glass slides with Vectashield Vibrance Antifade Mounting Medium and stored overnight in the fridge before imaging.

### 2.14 Confocal Microscopy

Slides were stored overnight in a fridge at 4°C before imaging to allow the mounting medium to reach its optimal refractive index. Slices were imaged on a Zeiss 900 inverted confocal microscope using 40×/1.3 NA or 63×/1.4 NA oil immersion objectives. Slices were imaged as tile scans with 10% overlap and 16× averaging.

### 2.15 Real‐Time Fluorescence Imaging

For combined circadian fluorescence imaging, SCN slices were sealed in glass‐bottom imaging dishes (P35G‐0‐10‐C, Mattek) containing the same HEPES‐buffered DMEM‐based recording medium as in the PMT recordings. Slices were imaged on an LV200 Imaging System (Olympus). Fluorescence channels were acquired sequentially, with acquisition set at 100 ms. The acquisition interval for circadian imaging was 30 min.

### 2.16 Pharmacological Treatments

A2ti‐1 (2‐(4‐phenyl‐5‐o‐tolyloxymethyl‐4H‐[1,2,4]triazol‐3‐ylsulfanyl) acetamide) and A2ti‐2 (2‐[4‐(2‐ethylphenyl)‐5‐o‐tolyloxymethyl‐4H‐[1,2,4]triazol‐3‐ylsulfanyl] acetamide) (MedChemExpress) were solubilized in DMSO to 100 mM. DMSO was used as the control vehicle treatment at a final concentration of 0.1%–0.2% dependent on experimental conditions. Dilutions for dose response curves were made in DMSO so that the concentration of DMSO across treatments was consistent. Pharmacological treatments were washed out by three serial transfers of the membrane and SCN into fresh medium for 10 min each step, before the final transfer to fresh medium for recording.

### 2.17 Analysis of Real‐Time Bioluminescent and Fluorescent Imaging

PMT recordings were analyzed by identification of peaks and troughs in the raw bioluminescence signal, as described previously (Patton, Morris, et al. 2023). The peak‐to‐peak period was calculated as the time difference between consecutive peaks and the period of an experimental interval was the mean of the peak‐to‐peak periods for that interval. The change in period (ΔPeriod) was the difference in hours between the mean period for the baseline and treatment intervals. Cycle amplitude was the absolute difference between trough and peak, normalized to the amplitude of the cycle before treatment (i.e., the last baseline peak and trough). Phase shifts were calculated as the time difference between the second peak post‐treatment and the predicted second peak post‐treatment, as extrapolated from the last pre‐treatment peak and baseline period. The second cycle post‐treatment accounts for any potential changes in waveform on the first cycle, therefore representing the sustained shift. Statistical analyses for cycle amplitude and phase‐shift meta‐analyses across experiments were carried using a linear mixed model approach where the main factors were treatment phase and treatment, and slice identity was a random effect to account for repeated treatments at different phases.

Relative amplitude error (RAE) values (an inverse measure of oscillation coherence) were determined using FFT‐NLLS measures in BioDARE2 (Zielinski et al. 2014) (https://biodare2.ed.ac.uk/). RAE values for treatments were normalized to the baseline. Washout dynamics were calculated by taking the ratio of the amplitude of the rising phase of the cycle (the amplitude between the preceding trough and the peak, Before [B]) and the amplitude of the falling phase (the amplitude between the peak and the following trough, After [A]). This ratio was termed the B/A ratio and was defined at steady state as the second peak post‐washout.

Real‐time fluorescence data were processed in FIJI (Schindelin et al. 2012) as described previously (Patton, Morris, et al. 2023). Fluorescence stacks were background‐subtracted using the built‐in rolling ball algorithm at 2 pixels before taking aggregate raw mean gray values. Data were detrended by subtracting a line fit to the last 72 h of the baseline interval of the recording projected forward into the treatment interval. Finally, smoothing was applied by a 5‐point (2.5 h) moving average. To see the general trend across experiments on pharmacological treatment, slices were temporally pseudo‐aligned by the final jRCaMP1a peak in the baseline interval to create a mean oscillation profile. Individual data points around the treatment interval were excluded from this aggregate if they did not align with other slices due to treatment artifact, thereby creating a gap between the final point in the baseline and the first point during treatment. Complete traces with the missing datapoints that underlie these are provided in Figure S3.

### 2.18 Experimental Design and Statistical Analysis

Where possible, slices received paired treatments (vehicle and drug) and were exposed to all concentrations along a dose–response curve (in an interleaved design). Where this was not possible (or slices died), slices were randomly assigned to groups. In all real‐time experiments, slice recordings covered baseline, treatment and (where possible) washout intervals. Baselines acted as additional internal paired controls alongside vehicle. All statistical tests used are listed in the text and were performed in Graphpad Prism with the exception of Rayleigh tests and Linear Mixed Model analyses which were carried out in R using the *r.test* function from the CircStats package (version 0.2‐6) (Lund and Agostinelli 2018) and the *lme* function from the *nlme* package (version 3.1‐162) (Pinheiro and Bates 2000; Pinheiro et al. 2024) alongside the emmeans package (version 1.10.2) (Lenth 2024), respectively.

All data were analyzed in Excel (Microsoft), R (R Foundation for Statistical Computing, Vienna, Austria. R version 4.3.0 [2023‐04‐21]), RStudio (Posit PBC, software version 2023.03.1+446), FIJI (Schindelin et al. 2012), and GraphPad Prism 10 (GraphPad). Statistical tests used are listed in the text and figure legends. All numbers reported in text are mean ± SEM unless otherwise stated. Scripts to reproduce R analyses are reproduced in Supporting Information.

## 3 Results

### 3.1 Assembly of a Circadian Proteome in the SCN

To define the circadian proteome of free‐running SCN tissue, we used SILAC‐MS. Through comparison of peptidergic MS spectra from a labeled “spike” reference control standard and unlabeled circadian samples (Figure 1A), and subsequent bioinformatic analysis, we ultimately identified 3182 SCN proteins. This *identified* proteome was refined further by applying the criterion that two of the three replicates from each circadian time point should provide reliable quantification of the protein. Consequently, 2082 proteins constituted the *quantified* proteome of SCN slices sampled over a full circadian cycle (Figure S1, Supporting Information S1). Quantified proteins were tested for significant (*p* < 0.05) circadian regulation by two parallel approaches. First, the RAIN‐algorithm (Thaben and Westermark 2014) was applied, producing a proteome of 304 rhythmic proteins (~15% rhythmic). Second, the JTK‐algorithm (Hughes et al. 2010) was applied, producing a proteome of 166 rhythmic proteins (~8% rhythmic). We generated a *circadian* proteome where the methods overlapped, representing 153 proteins from RAIN (~50%) and all but 13 JTK‐identified proteins (~92%). This circadian proteome constituted ~7% of the quantified proteome (Figure S1, Supporting Information S2). Clustering and visualization of the circadian proteome by raster plot revealed approximately equal phase distribution of peak protein expression throughout the circadian cycle (CT0/24 24%, CT6 21%, CT12 25%, and CT18 31%) (Figure 1B). This indicates that identification of the final circadian proteome did not result in any waveform bias.

Having assembled an SCN circadian proteome, we then performed bioinformatic analysis to reveal enriched processes, thereby pinpointing potential contributors to SCN circadian function. We focused on Gene‐Ontology terms (GO‐terms), specifically those that describe Molecular Function (MF). The top MF GO‐terms in our circadian proteome were related to actin, trafficking and transport, and cellular signaling pathways (Figure 1C, Supporting Information S3). These GO‐terms indicated that SCN cellular homeostasis is under circadian control. Further examination of enrichment of proteins related to the “calcium dependent‐protein binding,” “GTPase binding,” “ATP‐dependent protein binding,” and “S100 protein binding” indicated discrete cellular signaling pathways that may form inputs and outputs within the SCN network are also under circadian control.

To characterize this further, we focused on expression profiles of representative proteins. First, the synaptic protein Nsf, identified in a previous, smaller SCN proteome (Deery et al. 2009) was a circadian protein peaking around CT12 alongside the functionally related proteins Snap25 and Snap47 (Kadkova et al. 2019), which shared a common temporal profile (Figure 1D, Synaptic proteins). We then investigated profiles of representative proteins within other significant GO‐terms. Proteins involved in actin binding, encompassed by the two most‐significant GO‐terms “actin binding” and “actin filament binding” had staggered profiles: Gsn peaked sharply during the day at CT6 while, in contrast, Syne2 which binds actin filaments to the nuclear membrane and Fscn1 which bundles actin filaments (Adams 2004) (two potentially related functions) peaked at CT24/0 (Figure 1D, Actin binding). Together, these proteins suggest circadian dynamics to actin remodeling in the SCN as has been shown in fibroblasts (Hoyle et al. 2017). Next, we looked at proteins contributing to the “transmembrane transporter binding,” which may regulate SCN network activity (Patton, Morris, et al. 2023). The three representative proteins in this group: Arrb1, Fhl1, and Fkbp1a displayed differently phased circadian profiles, suggesting differential roles within the SCN clock. Arrb1, peaking at CT6, has been implicated in met‐Enk signaling where, in response to delta‐opioid receptor activation, it translocates to the nucleus to activate the *Per2* promoter (Bigliardi et al. 2024) (Figure 1D, Transmembrane Transporter Binding). Accordingly, met‐Enk signaling in the SCN modulates light induced phase‐shifts (Tierno et al. 2002). Finally, we assessed the nucleocytoplasmic transporters (Figure 1D, Nucleocytoplasmic carrier activity). This included Tnpo, Xpo1, and Xpo5, whose knockdown alters circadian period in cell models (Korge et al. 2018). Strikingly, loss of Tnpo shortened circadian period by reducing the rate of Per1 accumulation in the nucleus (Korge et al. 2018). Consistent with this role, Tnpo peaks at CT6 coincident with when Per proteins are accumulating. Nevertheless, the phase staggering of Xpo1 and Xpo5 relative to each other and to Tnpo suggests that they play different roles in clock function (Figure 1D, Nucleocytoplasmic carrier activity). Collectively, the differential temporal dynamics of these proteins indicate that even when their biological function is similar, they can occupy different temporal niches.

### 3.2 Identification of SCN Cellular Components Enriched in the Circadian Proteome

**FIGURE 2:**
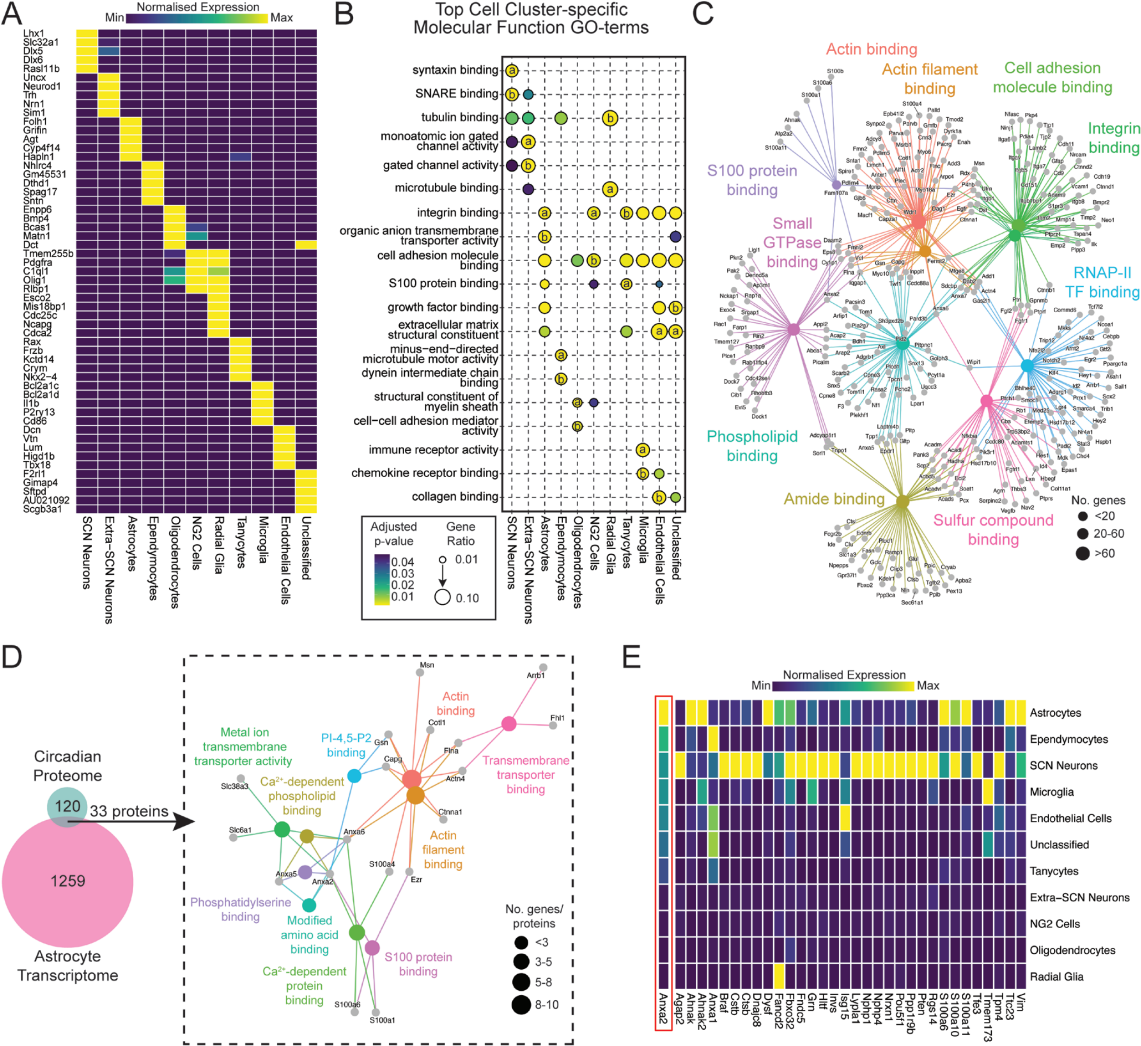
Transcriptomic analysis of the SCN reveals annexin‐A2 as an astrocyte‐enriched circadian protein. (A) Heatmap showing the top five genes identified in each cluster generated from a previously published scRNA‐seq dataset for free‐running SCN explants (Morris et al. 2021). (B) Dot‐plot showing the top two MF‐GO terms for each cluster in the scRNA‐seq dataset, showing the enrichment for the GO‐term (gene ratio) as dot size and adjusted *p*‐value as shading. The dots are labeled as “a” and “b” to denote the first and second top MF‐GO terms identified for that cell‐type, respectively. (C) A category network plot for the astrocytic transcriptome extracted from the scRNA‐seq data showing the top 10 most significant MF‐GO terms and the genes in the dataset that contribute to them, arranged as an interaction network. (D) Combination of the circadian proteome with the astrocyte transcriptome results in an overlap of 33 proteins/genes that are circadian in our dataset and arise from astrocytes. A category network plot for this overlap showing the top 10 most significant MF‐GO terms and the astrocytic proteins/genes in the dataset that contribute to them, arranged as an interaction network. Note that Anxa2 forms a nexus point in this network between multiple MF‐GO terms. (E) Heatmap showing cell‐type enrichment of transcripts that encode known interaction partners of Anxa2 within the scRNA‐seq cell clusters (derived from BioGRID database; Oughtred et al. 2021; Stark et al. 2006). Cell‐types (rows) are ordered by the enrichment of Anxa2 (bordered in red).

The cellular SCN network is heterogeneous, including neurons and astrocytes (Morris et al. 2021; Wen et al. 2020; Xu et al. 2021). Our SCN circadian proteome gave us the circadian profile of SCN proteins but lacked the cellular resolution to identify where in the network these proteins were expressed. To map our proteomic dataset to particular cell‐types, we utilized a published single‐cell transcriptomic dataset produced from similar free‐running SCN slices (Morris et al. 2021). We first clustered this dataset before manually annotating the cell‐types of clusters based on their top identifying genes. This allowed us to identify cell‐types (Figure 2A) with the transcriptomic profiles (defined previously; Morris et al. 2021; Wen et al. 2020) of: SCN neurons (*Slc32a1*, *Nms*, *Avp*, and *Vip*), extra‐SCN neurons (*Slc17a6*, *Th*, *Sst*, and *Agrp*), astrocytes (*Gfap*, *Aldh1l1*, *Aqp4*, and *Sox9*), ependymocytes (*Tmem212* and *Tctex1d4*), oligodendrocytes (*Mog* and *Plp1*), NG2‐cells (*Pdgfra*), radial glia (*Ccnb1* and *Ube2c*), tanycytes (*Col23a1*), microglia (*Ly86*, *C1qa*, and *Hexb*), endothelial cells (*Lum* and *Dcn*), and remnant unclassified cells (exclusion of other markers).

Having clustered SCN cell‐types via transcript expression, we applied an unbiased bioinformatic approach across the cellular clusters to determine whether any cell‐types aligned with the MF GO‐terms from the proteomic analysis. Analyzing the defining 250 genes in each cluster (Supporting Information S4), we plotted the top 2 GO‐terms for each cell‐type, resulting in 19 (partially redundant) examined GO‐terms (Figure 2B). Of interest, only one of the top cell‐type GO‐terms was also found in the circadian proteome: “S100 protein binding,” which to declining degrees of significance was specific to astrocytes, tanycytes, microglia and endothelial cells. Moreover, the astrocytes and tanycytes (two related glial‐cell types; Clasadonte and Prevot 2018) showed more significant enrichment. Given that astrocytes are known to actively participate in SCN network‐level timekeeping (Brancaccio et al. 2017, 2019; Patton, Morris, et al. 2023; Patton et al. 2022), we focused on them. When GO‐term analysis was applied to astrocytes independently of all other cell types, “S100 protein binding” was the fifth most significantly enriched MF GO‐term (Figure 2C, Supporting Information S5, adjusted *p*‐value < 0.0001), thereby describing one of the defining transcriptomic features of SCN astrocytes.

Having identified astrocytes as the cell‐type enriched in “S100 protein binding” related transcripts, we looked to combine our two‐omics data sets by comparing proteins identified as circadian (153) with astrocyte enriched transcripts (1292). This revealed 33 circadian proteins with transcripts enriched in the astrocytic transcriptome (Figure 2D). We then carried out our analysis on these 33 proteins/transcripts with “S100 protein binding” being the third most significant MF GO‐term (Figure 2D, Supporting Information S6). Between the two datasets, this was driven by the presence of S100 protein family members (S100a1, S100a4, and S100a6) and their interaction partners (Anxa2 and Ezr) (Figure 2D). Interestingly, annexin‐A2 (Anxa2) appeared as a nexus between seven of the top 10 MF GO‐term nodes, contributing to enrichment of: “S100 protein binding,” “metal‐ion transmembrane transporter activity,” “calcium‐dependent protein binding,” “calcium‐dependent phospholipid binding,” “phosphoinositol‐(4,5)‐bisphosphate (PI (4,5)P_2_) binding,” “phosphatidylserine binding,” and “modified amino acid binding” (Figure 2D).

To explore the possibility that Anxa2 and its interactors may be important to SCN astrocytic circadian function, regardless of their presence in the circadian proteome, we next assayed the cellular transcript expression of *Anxa2* and all of its interaction partners, as defined by BioGRID (Oughtred et al. 2021; Stark et al. 2006) (version 4.4). This highlighted 34 potential genes, including three members of the S100 protein family (*S100a6*, *S100a10*, and *S100a11*) that could encode known interaction partners of Anxa2. When plotted as a heatmap, although some of these proteins were restricted to other SCN cell‐types, only about a third of the potential interaction partners were enriched in astrocytes alongside *Anxa2*, positioning them as the cellular site of *Anxa2* action within the SCN and narrowing the pool of potential interactors (Figure 2E).

### 3.3 Identification of a Putative Anxa2‐S100a10 Interaction Within SCN Astrocytes

**FIGURE 3:**
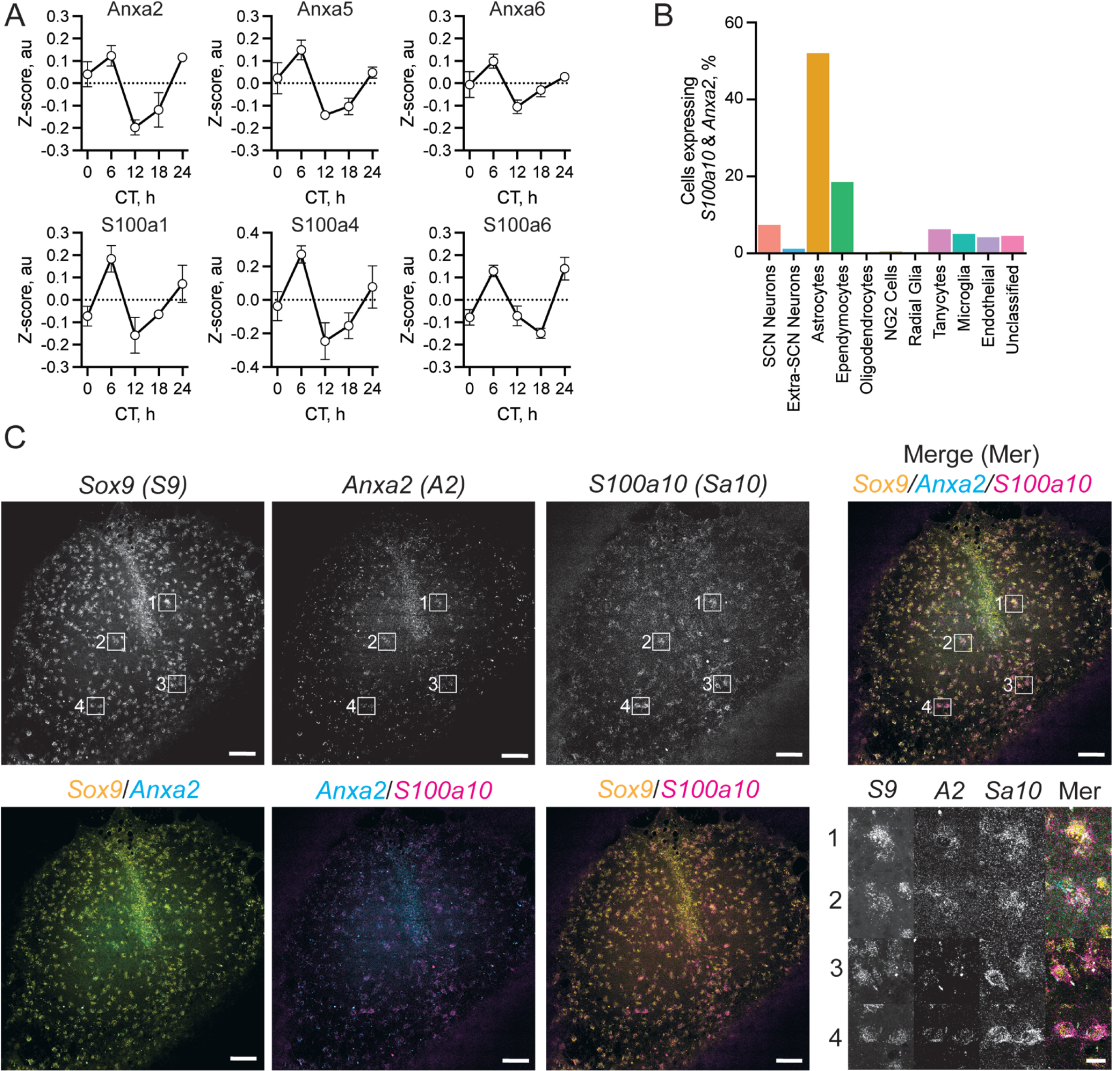
Annexin and S100 proteins share the same temporal profile and are co‐expressed in SCN astrocytes. (A) Plots of the circadian dynamics of abundance for Anxa (upper) and S100 (lower) proteins identified in the circadian proteome (Anxa2, 5, and 6 and S100a1, 4, and 6). (B) Histogram showing the % of cells in each cell‐type that co‐express Anxa2 and S100a10 transcripts, as calculated from scRNA‐seq data. (C) HCR in situ hybridization showing co‐registration of *Sox9* (*S9*), *Anxa2* (*A2*), and *S100a10* (*Sa10*) transcripts within SCN explants. Single grayscale images are shown on the upper line of panels along with a three‐color merge (Merge). Dual color merges are shown on the lower line of panels (left), alongside high magnification insets (right) corresponding to the white numbered boxes in the upper panels. Scale bars are 100 μm/20 μm.

As the Anxa and S100 families were identified as important proteins/genes at both proteomic and astrocytic transcriptomic levels, we assessed the temporal protein expression profiles of these family members. Anxa2, 6, and 7 were present in our proteome, along with S100a1, 4, and 6 (Figure 3A). Interestingly, these all showed the same circadian profile, peaking in the middle of the circadian day at CT6 despite only S100a6 being a potential interaction partner (Figures 2E and 3A). To assess a functional role for Anxa2 in the SCN astrocytes, we then re‐examined our pool of potential interaction partners (Figure 2E). Among *Anxa2* interaction partners enriched within the SCN astrocytes, co‐expression of *Anxa2* and *S100a10* stood out due to previous work implicating them as partners controlling a diverse range of cellular processes including exocytosis, ion channel translocation and modulation, and histone modification (Bharadwaj et al. 2021). Indeed, this interaction is so strong that S100a10 protein stability is dependent on interaction with Anxa2 (Puisieux et al. 1996). Although S100a10 was in our identified proteome, it was not quantified due to our initial stringent quality control.

To validate SCN expression of *S100a10*, and to show that *Anxa2* and *S100a10* are expressed in the same cells (and thereby positioned to interact), we queried the transcriptomic data for cellular co‐expression of these transcripts. Out of 29,822 cells across the transcriptomic data, we found that 18,969 cells expressed neither *S100a10* nor *Anxa2* with the remaining cells expressing either *Anxa2* or *S100a10* alone (1459 and 6745, respectively). Only 2649 cells co‐expressed *Anxa2* and *S100a10* together, and the majority of these were astrocytes (Figure 3B), with a smaller representation from ependymocytes. Given that half (50.7%) of the astrocytes co‐expressed *Anxa2* and *S100a10* in the scRNA‐seq dataset, we performed in situ hybridization on SCN slices to confirm this astrocytic co‐expression, using *Sox9* as a specific astrocytic marker (Endo et al. 2022; Sun et al. 2017). Importantly, these three transcripts co‐localized, demonstrating *Anxa2* and *S100a10* co‐expression in individual SCN astrocytes (Figure 3C, Figure S2).

### 3.4 Manipulation of the Anxa2‐S100a10 Interaction in SCN Explants Acutely Disrupts Astrocyte Function

Cellular translocation of Anxa2 to cell membranes is Ca^2+^‐ and S100a10‐dependent, being mediated by the formation of a hetero‐tetramer (Svenningsson and Greengard 2007): A2–A10. A2–A10 is Ca^2+^‐sensitive, and among other roles recruits and modulates various ion channels at the cell membrane, possibly to regulate ion flux (Gerke et al. 2005). This therefore positions the A2–A10 complex both as a potential sensor and as a regulator of cellular calcium levels ([Ca^2+^]_i_). Formation of the complex relies on accommodation of the N‐terminus of Anxa2 into a hydrophobic pocket on S100a10, an interaction that can be pharmacologically disrupted by the compound A2ti‐1, which binds to this pocket, preventing hetero‐tetramerization (Reddy et al. 2012). It is therefore a highly potent and specific inhibitor of A2–A10 association and so we used it to disrupt A2–A10 in SCN slices and thereby explore the contribution of A2–A10 to SCN circadian function.

**FIGURE 4:**
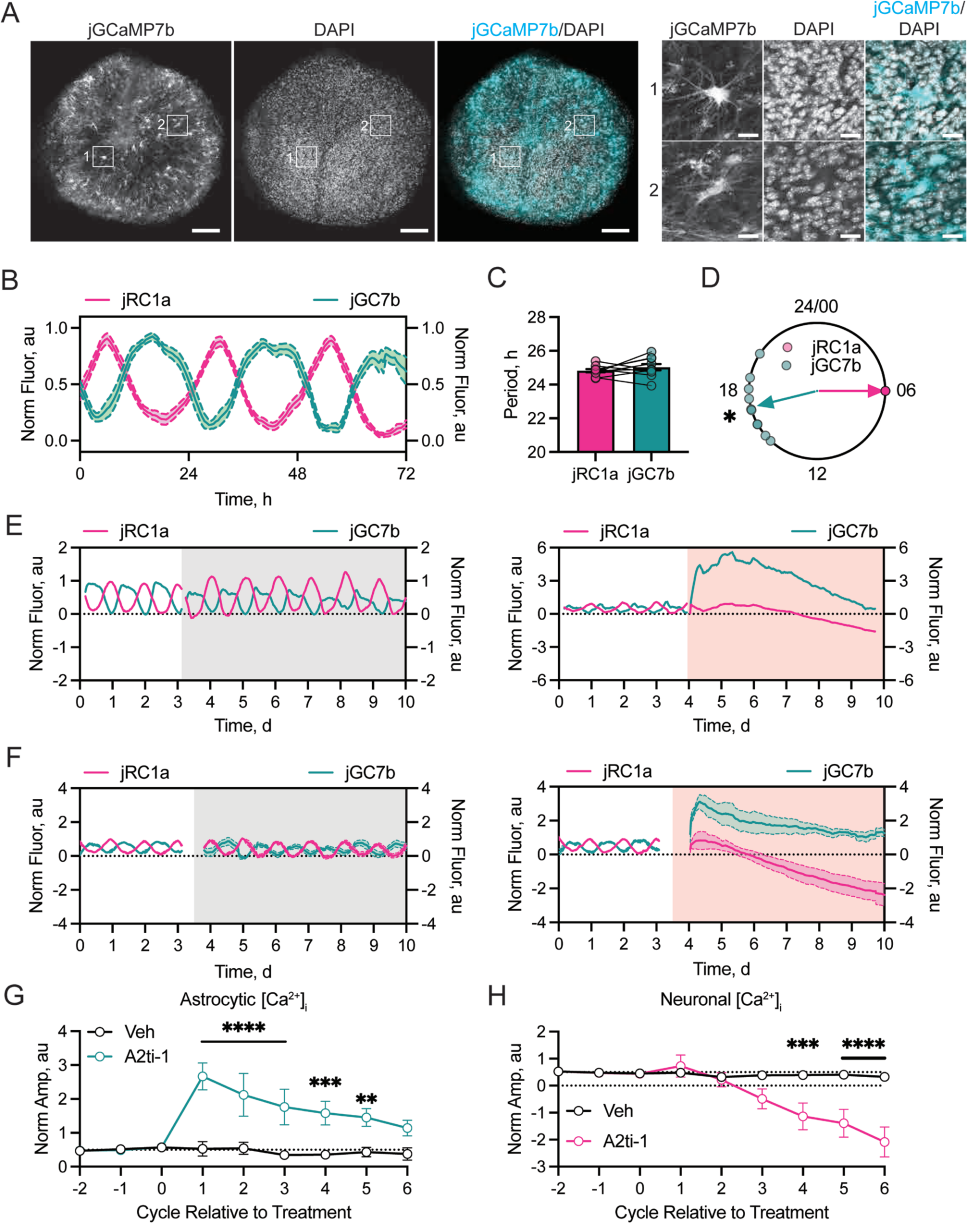
Disruption of the A2–A10 complex acutely elevates astrocytic [Ca^2+^]_i_. (A) Example confocal image of an SCN explant transduced with *GFAP*.jGCaMP7b AAV. Greyscale panels for native jGCaMP7b fluorescence (left) and DAPI (middle) alongside colored merge (right, Merge). The insets on the right show astrocytic morphology for the numbered boxed regions. In the merges, jGCaMP7b fluorescence is false‐colored as cyan. Scale bars are 100 μm/20 μm. (B) Example normalized average fluorescence traces from SCN showing the phasing of astrocytic [Ca^2+^]_i_ reported by jGCaMP7b (jGC7b, teal) alongside neuronal [Ca^2+^]_i_ reported by jRCaMP1a (jRC1a, pink). The first jRCaMP1a peak (CT06) for each slice was used to align the slices in circadian time. (C) Summary data showing the mean period for each reporter in a multiplexed recording. (D) Circular Rayleigh plot showing the relative phasing of astrocytic (jGC7b, teal) [Ca^2+^]_i_ across different slices relative to neuronal [Ca^2+^]_i_ (jRC1a, pink), which was set as CT06 for each slice. The direction of the arrows indicates the mean phase of the report. (E) Representative example normalized traces of jRCaMP1a (jRC1a, pink) and jGCaMP7b (jGC7b, teal) fluorescence from an SCN slice, before (unshaded) and during (shaded) treatment with vehicle (left, gray) and 80 μM A2ti‐1 (right, red). (F) Example normalized mean jRCaMP1a (jRC1a, pink) and jGCaMP7b (jGC7b, teal) traces of slices before (unshaded) and during (shaded) treatment with vehicle (left, gray) or 80 μM A2ti‐1 (right, red). Individual slices were registered in circadian time by alignment of the final jRCaMP1a peak of the baseline interval before treatment to generate group mean profiles under treatment. (G, H) Quantification of the effects of treatment on the individual reporters for jGCaMP7b (astrocytic [Ca^2+^]i) (G) and jRCaMP1a (neuronal [Ca^2+^]i) (H) calculated as normalized signal integrated over 24 h intervals for each slice. Vehicle treatment is shown by black lines, A2ti‐1 treatment is shown by colored lines. In (B, F) lines and shading represent mean ± SEM. In (C, D) points represent individual slices and where possible, lines are shown to indicate pairing of measures. In (G, H) points represent mean ± SEM. Statistics: D: Rayleigh test, *R* = 0.92, *p < 0.0001; (G, H) repeated measures two‐way ANOVA with Šidák's multiple comparisons test: *p = 0.04, **p = 0.004, ***p < 0.0008, ****p < 0.0001. Group sizes are (B–D) = 10; (F–H) = 6/6, Veh/A2ti‐1.

The first target of interest was [Ca^2+^]_i_ in SCN astrocytes, which reflects cellular activity and is under circadian control, peaking in circadian night when neuronal [Ca^2+^]_i_ is low (Brancaccio et al. 2017). To monitor [Ca^2+^]_i_ in astrocytes, we expressed the calcium reporter jGCaMP7b under control of the astrocyte‐specific promoter *GFAP* via AAV vector (Lohr et al. 2021). Confocal microscopy confirmed jGCaMP7b signal localized to SCN astrocytes, as evidenced by the characteristic stellate cellular morphology (Figure 4A). Slices were also transduced with AAV *Syn*.jRCaMP1a to monitor neuronal [Ca^2+^]_i_. [Ca^2+^]_i_ levels in astrocytes and neurons were both highly circadian (Figure 4B), with common periods of 25.0 ± 0.2 and 24.8 ± 0.1 h, respectively (paired two‐tailed *t*‐test *t* (9) = 1.04, *p* = 0.32, Figure 4C) but different phases: astrocytic and neuronal peaks were ~12 h apart, as reported (Brancaccio et al. 2017). We have shown previously that neuronal [Ca^2+^]_i_ peaks at ~CT6 (Patton, Morris, et al. 2023), which provided a phase reference that assigned the peak of the astrocytic [Ca^2+^]_i_ rhythm to CT17.1 ± 0.5 h (Rayleigh test, *R* = 0.92, *p* < 0.0001, Figure 4D), again confirming the astrocytic origin of the signal.

We then treated reporter‐expressing slices with vehicle or 80 μM A2ti‐1 (Figure 4E,F, Figure S3). As expected, vehicle treatment had no effect on the oscillations, whereas 80 μM A2ti‐1 markedly disrupted the rhythms in [Ca^2+^]_i_ in both cell types (Figure 4E,F). Treatment with A2ti‐1 caused an immediate and sustained elevation of astrocytic [Ca^2+^]_i_, (repeated‐measures two‐way ANOVA: treatment effect *F* (1, 5) = 16.34, *p* = 0.01; time effect *F* (8, 40) = 8.41, *p* < 0.0001; interaction *F* (8, 40) = 6.10, *p* < 0.0001) (Figure 4G). In contrast, neuronal [Ca^2+^]_i_ was suppressed by A2ti‐1 (repeated‐measures two‐way ANOVA: treatment effect *F* (1, 5) = 13.97, *p* = 0.018; time effect *F* (8, 40) = 10.5, *p* < 0.0001; interaction *F* (8, 40) = 9.13, *p* < 0.0001) (Figure 4H). Compared to the rapid effect seen in astrocytes, however, the suppression of neuronal [Ca^2+^]_i_, was progressive, moving out of the operating range of vehicle treatment only after 4 cycles (Figure 4H). In both cell‐types, the circadian regulation of [Ca^2+^]_i_ was rapidly (1–2 cycles) damped, notwithstanding the overall elevation or decline. Thus, blockade of formation of the astrocytic A2–A10 complex disrupted SCN timekeeping across the cellular network, suspending circadian oscillations and functionally pushing astrocytic and neuronal calcium‐reported metabolic states away from one another and to their respective “night‐time” states.

### 3.5 Manipulation of the Anxa2‐S100a10 Interaction in SCN Explants Disrupts Network Circadian Timekeeping

**FIGURE 5:**
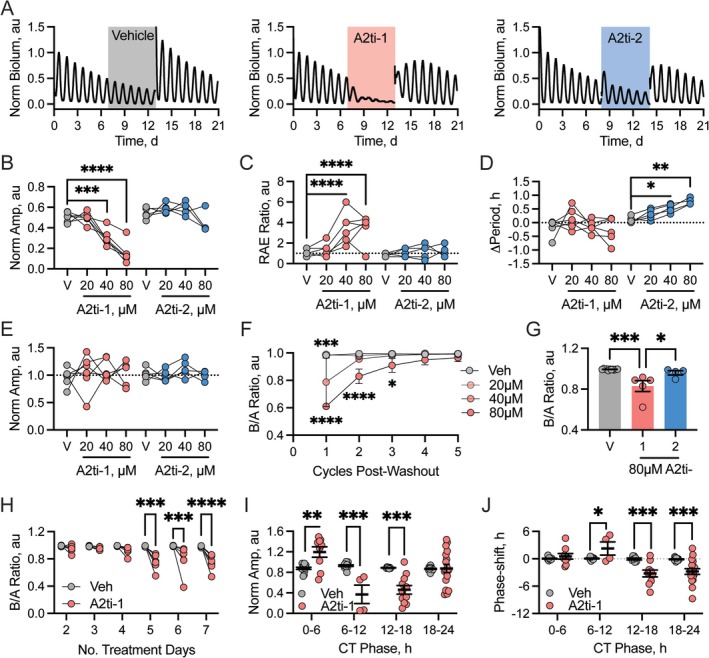
Disruption of the A2–A10 complex compromises SCN circadian timekeeping. (A) Representative normalized Per2::Luc bioluminescent traces from SCN treated with vehicle (left), 80 μM A2ti‐1 (middle) or 80 μM A2ti‐2 (right). Shading indicates treatment, bounded by pre‐treatment and post‐treatment washout intervals. (B–D) Summary data showing normalized amplitude change (B), relative amplitude error (RAE) (C), and period change (Δperiod) (D), during treatment with vehicle (gray, V) or different doses of A2ti‐1 (red) and A2ti‐2 (blue), relative to pre‐treatment. (E) Amplitude recovery of oscillation after the termination of treatment (washout). (F) Quantification of A2ti‐1 washout dynamics showing asymmetric increases in oscillation amplitude over successive cycles. (G) Comparison of washout dynamics following vehicle (V, gray), A2ti‐1 (1, red), and A2ti‐2 (2, blue). (H) Washout dynamics of slices following different intervals of treatment with vehicle (gray circles) or 80 μM A2ti‐1 (red circles). (I) Meta‐analysis of the acute effect of vehicle (gray circles) or 80 μM A2ti‐1 (red circles) on rhythm amplitude, arranged by phase of treatment. (J) Meta‐analysis of the phase‐shifting effect of vehicle (gray circles) or 80 μM A2ti‐1 (red circles), arranged by phase of treatment. In (B–E) and (G–J) individual points represent individual SCN slices. In (F) individual points represent mean ± SEM. In (G) bars represent mean ± SEM and in (I, J) lines represent mean ± SEM. Points connected by a line represent paired measures. Statistics: (B–E) Mixed effects two‐way ANOVA followed by Dunnett's multiple comparisons test: *p < 0.05, **p < 0.01, ***p < 0.001, ****p < 0.0001; (F) mixed effects two‐way ANOVA followed by Dunnett's multiple comparisons test: *p = 0.03, ***p = 0.0009, ****p < 0.0001, calculated by comparison against vehicle at different time points (80 μM vs. vehicle comparison symbols are shown below the plot, 40 μM vs. vehicle are shown above the plot); (G) mixed effects one‐way ANOVA followed by Tukey's multiple comparisons test: *p = 0.014, ***p = 0.0003; (H) repeated‐measures two‐way ANOVA with Šidák's multiple comparisons test: ***p < 0.0008, ****p < 0.0001; (I, J) linear mixed model with Šidák's multiple comparisons test: *p = 0.02, **p = 0.001, ***p < 0.0003. Group sizes are (B–G): 6/5 A2ti‐1/A2ti‐2 treated SCN explants, (H) 6/6 Veh/A2ti‐1; (I) CT0‐6 14/9, CT6‐12 14/4, CT12‐18 7/11, CT18‐24 12/18 Veh/A2ti‐1; (J) CT0‐6 14/9, CT6‐12 14/4, CT12‐18 7/10, CT18‐24 12/18 Veh/A2ti‐1.

Given the immediate disruption of astrocytic [Ca^2+^]_i_ levels and delayed neuronal disruption, we then tested the effect of A2ti‐1 on overall SCN network time‐keeping using the Per2::Luciferase (Per2::Luc) translational reporter of the TTFL. To confirm specificity of A2ti‐1 effects, we used a second compound, A2ti‐2, which shares the chemical structure of A2ti‐1 but lacks an ethyl group. This leads to a 10‐fold reduction in potency (A2ti‐1 IC_50_ ≈ 24 μM; A2ti‐2 IC_50_ ≈ 230 μM) and so provides a valuable control treatment for any off‐target effects of A2ti‐1 in our tested concentration range (Reddy et al. 2012). Bioluminescence from SCN explants expressing Per2::Luc was recorded before and during chronic treatment with either vehicle, A2ti‐1 or A2ti‐2 at concentration ranges between 20 and 80 μM and also after wash‐out to monitor the reversibility of any effects (Figure 5A). Treatment with vehicle or A2ti‐2 had no obvious effect on the Per2::Luc circadian oscillation. In contrast, A2ti‐1 severely and dose‐dependently reduced the amplitude of the ongoing oscillation within 1 circadian cycle, down to 15% ± 4% of the pre‐treatment amplitude (Figure 5A,B) (mixed‐effects two‐way ANOVA: dose effect *F* (3, 26) = 24.67, *p* < 0.0001; treatment effect *F* (1, 9) = 49.65, *p* < 0.0001; dose × treatment interaction *F* (3, 26) = 9.67, *p* = 0.0002). To confirm the specificity of the compounds, we performed combined treatments with A2ti‐1 and A2ti‐2 and saw no blockade or enhancement of the suppression seen with 40 μM A2ti‐1 when 40 μM A2ti‐2 was also present (Figure S4). Accompanying the suppressed amplitude, A2ti‐1 also dose‐dependently reduced the quality of the ongoing oscillation (assessed by relative amplitude error [RAE]) (Figure 5C) (mixed‐effects two‐way ANOVA: dose effect *F* (3, 26) = 11.55, *p* < 0.0001; treatment effect *F* (1, 9) = 10.15; *p* = 0.01, dose × treatment interaction *F* (3, 26) = 7.49, *p* = 0.0009). Again, A2ti‐2 was without effect on the oscillation. It should be noted, however, that whereas either A2ti‐1 or vehicle treatment did not alter period, there was a dose‐dependent increase in period up to 0.77 ± 0.06 h with A2ti‐2 (Figure 5D) (mixed‐effects two‐way ANOVA: dose effect *F* (3, 26) = 2.58, *p* = 0.08; treatment effect *F* (1, 9) = 30.33, *p* = 0.0004; dose × treatment interaction *F* (3, 26) = 5.25, *p* = 0.006). We interpreted this period‐lengthening by A2ti‐2 as a potential off‐target effect of the drug.

These results show that pharmacological disruption of the astrocyte‐restricted A2–A10 complex had profound impact on SCN timekeeping, but importantly, was reversible, as evidenced by the return of high‐amplitude Per2::Luc rhythms following washout of the compound under all doses tested (Figure 5A,E) (mixed‐effects two‐way ANOVA: dose effect *F* (3, 34) = 0.69, *p* = 0.56; treatment effect *F* (1, 34) = 0.001; *p* = 0.97, dose × treatment interaction *F* (3, 34) = 0.19, *p* = 0.90). Interestingly, although the oscillation returned to expected levels, this was not immediate: there was a progressive increase in amplitude following withdrawal of A2ti‐1 (Figure 5A,F). We therefore explored this formally, using a ratio of the amplitude of the rising slope of the oscillation to that of the falling slope as a metric to assess progressive re‐establishment of the oscillation. This revealed significant differences in the re‐establishment of stable oscillation across the doses of A2ti‐1 compared to vehicle measurements (Figure 5F) (mixed‐effects two‐way ANOVA: cycle effect *F* (4, 20) = 15.55, *p* < 0.0001; dose effect *F* (3, 15) = 14.12; *p* = 0.0001; cycle × dose interaction *F* (12, 40) = 5.33, *p* < 0.0001). Following 40 μM A2ti‐1, the oscillation recovered completely after 2 cycles, whereas after 80 μM A2ti‐1, it took 4 cycles to recover. To examine this more clearly, we compared the ratios of the second peak post‐washout (to account for washouts where the preceding trough was missing) for slices after treatment with vehicle, 80 μM A2ti‐1 or 80 μM A2ti‐2 (Figure 5G) (mixed‐effects one‐way ANOVA, *F* (2, 17) = 12.63, *p* = 0.0004). As there was no significant difference between this measure for vehicle and A2ti‐2 (Tukey's multiple comparisons, vehicle vs. A2ti‐2, 0.99 ± 0.002 au vs. 0.96 ± 0.02 au, *p* = 0.61), this effect was linked to the amplitude recovery following washout of A2ti‐1 and not off‐target effects of the compound. We previously interpreted this progressive “winding up” of the oscillation following drug treatments targetted at astrocytes as a reflection of an iterative reciprocal reinforcement between the TTFLs of the astrocytes and neurons, each providing a progressively stronger signal as network recovery evolved. The current observations are therefore consistent with disruption of an astrocyte‐specific complex compromising network function, with its recovery in astrocytes on wash‐out followed by progressive re‐integration across the network (Patton, Morris, et al. 2023).

To put this recovery into context and probe the time‐course of network sensitivity to A2ti‐1, we performed further experiments in which slices were treated with A2ti‐1 for different durations ranging from 2 to 7 days before washout and then monitored the time‐course of recovery (Figure 5H). This revealed a significant effect of treatment length on the oscillation dynamics following washout (repeated‐measures two‐way ANOVA: treatment effect *F* (1, 30) = 52.00, *p* < 0.0001; treatment length effect *F* (5, 30) = 4.95, *p* = 0.002; interaction *F* (5, 30) = 4.61, *p* = 0.003) (Figure 5H). Furthermore, post hoc multiple comparisons testing revealed that the washout effect was only manifested once SCN were exposed to A2ti‐1 for more than 4 days (Figure 5H). This is consistent with the [Ca^2+^]_i_ data, whereby changes in neuronal [Ca^2+^]_i_ were delayed and moved significantly out of their operating range only after 4 cycles of treatment (Figure 4H). These washout dynamics indicate that progressive amplification of the oscillation is likely caused by a cycle‐to‐cycle reversal of the dissociation of neurons and astrocytes within the network.

This effect, driven specifically by disruption of astrocyte function, was confirmed by the phase‐dependent effect of A2–A10 hetero‐tetramer disruption on the acute amplitude of the PER2::Luc report (Figure 5I). Throughout our experiments, 80 μM A2ti‐1 treatment started at different circadian phases and so we performed a meta‐analysis of slices treated in dose–response and treatment length experiments. Analysis of this pooled data revealed bidirectional phase‐specific changes to the amplitude of the first peak post‐treatment (Linear Mixed Model: Treatment effect *F* (1, 51) = 3.95, *p* = 0.05; Phase effect *F* (3, 51) = 12.97, *p* < 0.0001; Interaction *F* (3, 51) = 14.37, *p* < 0.0001) (Figure 5I, Figure S5). Importantly, post hoc analysis revealed that this phase‐specific effect manifests as acute amplitude reductions when drug was applied at phases during which astrocytic [Ca^2+^]_i_ is rising or peaking (Figure 5I: CT6‐12 Veh 0.93 ± 0.01 au vs. A2ti‐1 0.37 ± 0.18 au, *p* = 0.0001; CT12‐18 Veh 0.89 ± 0.004 au vs. A2ti‐1 0.46 ± 0.08 au, *p* = 0.0002) while treatment during phases when astrocytic [Ca^2+^]_i_ is falling or at low levels either enhanced the following peak or was without effect (Figure 5I: CT0‐6 Veh 0.88 ± 0.02 au vs. A2ti‐1 1.20 ± 0.10 au, *p* = 0.001; CT18‐24 Veh 0.87 ± 0.01 au vs. A2ti‐1 0.88 ± 0.08 au, *p* = 0.96). This pattern of acute suppression of the amplitude of the oscillation is comparable with astrocyte‐specific disruption via the glial metabolic toxin fluorocitrate, where CT6‐18 is acutely sensitive to treatment (Patton et al. 2022).

Due to the observed phase‐dependence of changes in the TTFL amplitude, we examined whether A2ti‐1 treatment altered the phase of the ongoing oscillation (Figure 5J). Across these experiments, where we could accurately quantify the time of the second peak (88/89), we saw changes in phase that were specific to the phase of A2ti‐1 application (Linear Mixed Model: Treatment effect *F* (1, 50) = 9.96, *p* = 0.003; Phase effect *F* (3, 50) = 12.88, *p* < 0.0001; Interaction *F* (3, 50) = 9.96, *p* < 0.0001). These phase‐shifts were mainly phase delays occurring in circadian night, with a few advances in mid‐to‐late circadian day, as revealed by post hoc analysis (Figure 5J: CT0‐6 Veh 0.05 ± 0.08 h vs. A2ti‐1 0.54 ± 0.63 h, *p* = 0.54; CT6‐12 Veh 0.07 ± 0.08 h vs. A2ti‐1 2.27 ± 1.44 h, *p* = 0.02; CT12‐18 Veh −0.06 ± 0.21 h vs. A2ti‐1 −3.23 ± 0.75 h, *p* = 0.0003; CT18‐24 Veh −0.11 ± 0.10 h vs. A2ti‐1 −2.79 ± 0.61 h, *p* = 0.0001). This is the first evidence that direct manipulation of an endogenous astrocytic signaling pathway can alter the ongoing phase of the SCN TTFL oscillation (Patton et al. 2022), elevating the participation of astrocytes into a facet of SCN network level timekeeping beyond the modulation of period and/or amplitude.

## 4 Discussion

By a combination of proteomics and single‐cell transcriptomics of free‐running SCN slices, we identified Anxa2 and S100a10 as potential astrocytic circadian‐relevant interaction partners. Experimental disruption of their interaction elevated astrocytic calcium levels and suppressed neuronal activity, which ultimately compromised SCN‐wide TTFL oscillations. This highlights the critical nature of the A2–A10 interaction as a control point in SCN timekeeping and expands our understanding of how astrocytic circadian signals may integrate into, and direct, the neural timing networks of the SCN.

We used SILAC‐MS with labeled SCN tissue as reference rather than Neuro2A cells (Chiang et al. 2014) because the latter may not express the complete spectrum of SCN proteins. We found that ~7% of quantified proteins were circadian. Although this is below ~13% estimated from non‐SILAC methods (Deery et al. 2009) with less conservative circadian expression criteria (significant ANOVA), it nevertheless contains appreciably more circadian proteins overall (153 vs. 53). Furthermore, synaptic proteins again featured significantly, indicative of the molecular cycles that underpin the circadian rhythm of electrical activity of the SCN. A limitation of our approach, however, is that we applied a low frequency of sampling, that is, 6‐hourly. This may have limited the number of rhythmic proteins that we were able to identify. Nevertheless, combining this proteome with single‐cell transcriptomics (Morris et al. 2021) identified Anxa2 as an important astrocyte‐enriched protein. This was confirmed by in situ hybridization, where transcripts encoding *Anxa2* and its classical binding partner *S100a10* (Erikson et al. 1984; Gerke and Weber 1985a, 1985b) were enriched in SCN astrocytes alongside the astrocyte‐specific marker *Sox9* (Endo et al. 2022; Sun et al. 2017). This confirmed our bioinformatic analysis and positioned astrocytes as the cellular location of A2–A10 interaction within the SCN.

Anxa2 is a small Ca^2+^‐dependent protein implicated in cellular processes including endo‐ and exo‐cytosis, actin polymerization, and ion channel localization and modulation (Gabel et al. 2020; Okura et al. 2023; Seo and Svenningsson 2020). Importantly, these functions require it to undergo a mutually reinforcing interaction with S100a10 that stabilizes S100a10 and increases the affinity of Anxa2 for Ca^2+^ (Bharadwaj et al. 2021). Even though S100a10 is a member of the S100 Ca^2+^‐binding family of proteins, it is unique because changes in its EF‐hand domains render it insensitive to Ca^2+^. This locks it into a constitutively active conformation that is dependent on Anxa2 for calcium‐sensing (Madureira et al. 2012; Réty et al. 1999). Through the stabilizing interaction with Anxa2, S100a10 activity is controlled by the availability of Anxa2 (Okura et al. 2023) rather than by Ca^2+^, and formation of A2–A10 is integral to the function of both proteins (Bharadwaj et al. 2021). Hence, circadian expression of Anxa2 in the SCN has mechanistic relevance.

Alongside Anxa2, our proteomic screen also identified other members of the S100 protein family as circadian proteins in the SCN, including S100a1, 4, and 6 (Figure 3A). These other S100 proteins may be of interest, as they oscillate in phase with the co‐identified Anxa proteins and, in the case of S100a4 and 6, are potential interaction partners of Anxa2 and are enriched in SCN astrocytes (Figure 2E). Consistent with this, S100a4 and 6 are expressed in astrocytes (Kozlova and Lukanidin 1999; Yamashita et al. 1999) and their expression is induced in response to neuroinflammatory and neurodegenerative insults (D'Ambrosi et al. 2021; Filipek and Lesniak 2020), as is S100a10 (King et al. 2020; Li et al. 2019). Furthermore, a role for the interplay between the circadian clock of astrocytes and neurodegenerative phenotypes (McKee et al. 2020), is supported by the observation that transcript expression of *S100a6* is enhanced by *Bmal1* knockout (McKee et al. 2022), suggesting a role for the astrocytic TTFL in the regulation of circadian expression of these proteins. S100 proteins may therefore play further important roles in SCN astrocyte function at the circadian and network levels in both physiological and pathophysiological contexts.

Pharmacological disruption of A2–A10 is, arguably, preferable to genetic depletion due to the varied interaction partners and functions of Anxa2 and S100a10 (Gerke et al. 2024; Okura et al. 2023). Despite the potential for off‐target effects from pharmacological interventions, A2ti‐1 is specifically designed to disrupt the binding of the Anxa2 N‐terminus into the pocket on S100a10 in which it is normally accommodated. A2ti‐1 is almost as efficacious as the Anxa2 N‐terminal peptide itself (Reddy et al. 2011, 2012). Treatment with A2ti‐1 therefore better affords the possibility of disrupting A2–A10 without compromising the other functions of the individual proteins. Furthermore, the low affinity variant A2ti‐2 provides a useful control compound that does not enhance or block the effects of A2ti‐1 despite the structural similarity of the compounds. This observation links the amplitude effects to A2–A10 disruption (Figure S4). A2ti‐1 treatment therefore specifically reveals the role of the complex and not the individual proteins. Treatment of SCN explants with A2ti‐1 acutely elevated astrocytic [Ca^2+^]_i_, progressively suppressed neuronal [Ca^2+^]_i_ and significantly disrupted SCN network‐level timekeeping, with a damped TTFL. These effects point to the SCN being pushed into a permanent night‐like state, similar to that seen when extracellular GABA levels are chronically elevated (Patton, Morris, et al. 2023). The acute rise in astrocytic [Ca^2+^]_i_ implies a role for A2–A10 in lowering astrocytic [Ca^2+^]_i_, acting as a potential brake on astrocytic activity. Consistent with this, Anxa2 levels are highest in circadian day, coincident with low astrocytic [Ca^2+^]_i_ levels (Brancaccio et al. 2017). Importantly, A2–A10 formation is favored at this time because it is independent of Ca^2+^ whereas other Annexin/S100 interactions are Ca^2+^‐dependent (Okura et al. 2023) and would be expected to form in circadian night. Circadian control of Anxa2 abundance is therefore likely integral to its role in SCN astrocyte metabolism, but how the rhythm in protein levels is generated is unclear. In the SCN, *Anxa2* transcripts are not appreciably rhythmic by microarrays (Panda et al. 2002; Pizarro et al. 2013) and only marginally higher at night by scRNA‐seq (Morris et al. 2021) (log2FC = −0.58, adjusted *p* = 0.002; Figure S6). Circadian control of Anxa2 abundance and A2–A10 interaction could therefore be mediated post‐translationally (Babiychuk et al. 2002; Grindheim et al. 2017; Wang and Lin 2014).

A2–A10 controls many cellular processes, including the expression of cationic and anionic ion channels, as well as the metabotropic glutamate receptor mGluR5 (Seo and Svenningsson 2020; Svenningsson and Greengard 2007). It is therefore well positioned to modulate rapidly various intracellular environments and signaling pathways, the functions of which may be specific to cellular context. Interestingly, the knockout of the related astrocyte‐specific annexin Anxa7 disrupted [Ca^2+^]_i_ dynamics and homeostasis in primary cultured astrocytes (Clemen et al. 2003). Conversely, the overexpression of the related Anxa1 in MCF‐7 cells reduced the mobilization of Ca^2+^ from intracellular stores (Frey et al. 1999). Consistent with our observations, a conserved function of annexins may therefore be to sense as well as buffer [Ca^2+^]_i_ levels, potentially through modulation of Ca^2+^ influx or release from intracellular stores.

Disruption of A2–A10 within SCN astrocytes immediately and chronically pushed them into an active state, while neurons were progressively pushed to inactivity, and both cell‐types lost circadian [Ca^2+^]_i_, rhythms. At the network TTFL level, disrupting astrocytic A2–A10 had phase‐specific effects. First, the amplitude of Per2::Luc expression was acutely enhanced by treatment at CT0‐6 but suppressed between CT6‐18. Treatment between CT18‐24/0 had no overall effect on amplitude but showed large delays or advances in different slices, indicative of an unstable response. The phase variation may reflect the result of increasing astrocytic [Ca^2+^]_i_ when, spontaneously, it is either falling (CT18‐6) or rising (CT6‐18). The sensitivity of the SCN TTFL to suppression of astrocytic metabolism with the gliotoxin fluorocitrate is also greatest at CT6‐18 (Patton et al. 2022). Second, we observed that the phase of the remaining TTFL oscillation was shifted depending on the phase of A2–A10 disruption, with large phase delays when treated during circadian night (CT12‐24). These phase shifts were surprising, as direct chemogenetic activation of astrocytes does not alter SCN phase (Patton et al. 2022), although manipulation of astrocyte‐neuronal signaling can elicit SCN phase shifts in other contexts (Hablitz et al. 2020). This suggests that astrocytic control of SCN network function is more complex than simply the activation state of the cells and may rely on the magnitude and timing of the activation relative to other network components. This underlines the importance of the antiphasic circadian activity of the astrocytes and neurons in the SCN network (Smyllie et al. 2025).

Disrupting the timing of astrocytic network control during the day, either by preventing their uptake of GABA (Patton, Morris, et al. 2023) or allowing them to become active at a time when they are normally quiescent, has pronounced consequences for network‐level SCN timekeeping. Interestingly, this interaction is antagonistic: inhibitory drive from astrocytes via mechanisms that converge on GABA (Brancaccio et al. 2017; Patton, Morris, et al. 2023) more potently disrupts timekeeping than chronic neuronal activity (Patton, Morris, et al. 2023). Consistent with these observations, we have previously hypothesized that inhibitory signals from the astrocytes to the neurons explain why the astrocytic clock can efficiently slow down, but not accelerate network‐level oscillation (Patton et al. 2022). Despite this evidence for an inhibitory drive between astrocytes and neurons, there must also be positive reciprocal reinforcement between the two to complete the loop. Indeed, when A2–A10 disruption is reversed, we saw a progressive building of the amplitude of the recovering TTFL oscillation, indicative of a mutual reintegration and reinforcement of the astrocytes and neurons within the network. This progressive, rather than immediate, enhancement was also noted where SCN network function was disrupted by the compromise of GABA uptake by astrocytes, leading to direct suppression of neuronal activity (Patton, Morris, et al. 2023).

Our results raise the question of whether astrocytes in other brain areas act in the same way on GABAergic interneurons to modulate excitatory neuronal function within specific networks. In the SCN, A2–A10 controls astrocytic activity as indicated by localized co‐expression of *Anxa2* and *S100a10* transcripts (Figure S7, Wen et al. 2020). This may be a conserved astrocytic mechanism, as enrichment of both these transcripts occurs in astrocytes sampled from other areas including the spinal cord and motor cortex (Endo et al. 2022). Further analysis of whether this interaction functionally specifies a subpopulation of astrocytes is required, considering the high degree of inter‐ and intra‐regional heterogeneity of astrocytes (Endo et al. 2022; Farmer and Murai 2017; Stogsdill et al. 2023). This leads to three specific questions. First, how is astrocytic activity controlled by the Anxa2‐S100a10 interaction? Second, does this mechanism generalize to the inhibitory actions of astrocytes in other brain areas? And third, is this interaction and astrocytic network control in other brain areas regulated at the circadian (or sleep) level?

## Author Contributions

A.P.P., T.P.K., and M.H.H. designed the experiments. A.P.P., T.P.K., and E.S.M. performed the experiments. A.P.P. analyzed the data. N.J.S., E.L.M., and M.S. provided reagents, data, and expertise. A.P.P. and M.H.H. wrote the manuscript. All authors reviewed the manuscript.

## Conflicts of Interest

The authors declare no conflicts of interest.

## Supporting information


Data S1.

**Supporting Information 1** Quantified SCN Proteome.

**Supporting Information 2** Circadian SCN Proteome.

**Supporting Information 3** Circadian Proteome MF‐GO.

**Supporting Information 4** Cluster Transcripts MF‐GO.

**Supporting Information 5** Astrocyte Transcriptome MF‐GO.

**Supporting Information 6** Circadian Proteome & Astrocyte Transcriptome Overlap MF‐GO.

## Data Availability

The mass spectrometry proteomics data have been deposited to the ProteomeXchange Consortium via the PRIDE (Perez‐Riverol et al. 2025) partner repository with the dataset identifier PXD061617. Single‐cell RNA sequencing data reanalyzed in this manuscript (Morris et al. 2021) are publicly available from the NCBI Gene Expression Omnibus with the accession number GSE167927.
