# Supplementary material for: Multi‐Omic Analysis Reveals Astrocytic Annexin‐A2 as Critical for Network‐Level Circadian Timekeeping in the Suprachiasmatic Nucleus
Source: Glia. 2025 Apr 2;73(7):1483–501. doi: 10.1002/glia.70018 (PMC12121465; doi:10.1002/glia.70018)
Supplement: Supplementary file 1 — Data S1. [file GLIA-73-1483-s004.pdf]

Supplementary Material for

Multi-omic analysis reveals astrocytic Annexin-A2 as critical for network-level circadian timekeeping in the suprachiasmatic nucleus

Andrew P. Patton*, Toke P. Krogager, Elizabeth S. Maywood, Nicola J. Smyllie, Emma L. Morris, Mark Skehel and Michael H. Hastings*

Division of Neurobiology, Medical Research Council Laboratory of Molecular Biology, Cambridge, CB2 0QH, U.K.

*Authors for correspondence
Andrew P. Patton
Email: apatton@mrc-lmb.cam.ac.uk
Michael H. Hastings
Email: mha@mrc-lmb.cam.ac.uk

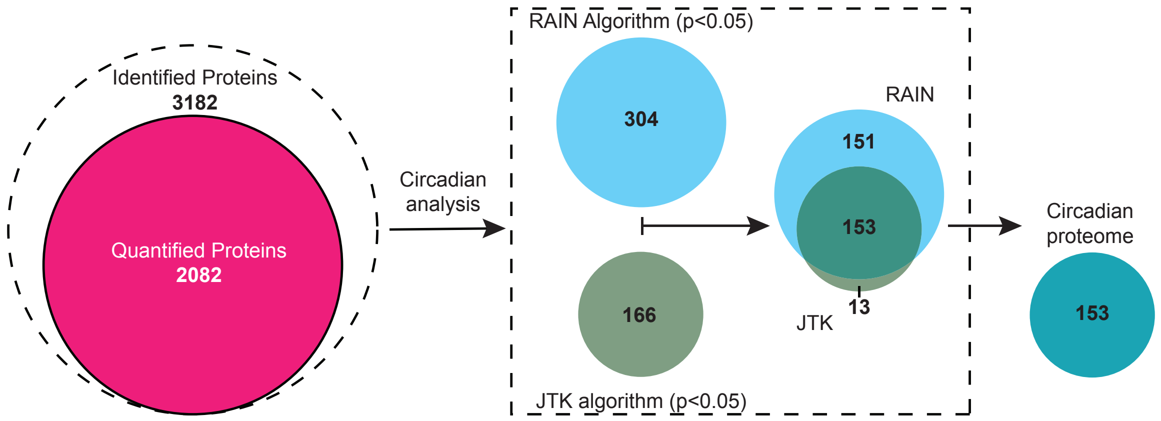

Supplementary Figure 1. Overlap of SCN circadian proteins identified with RAIN and JTK-cycle algorithms.

Venn diagrams showing the numbers of proteins identified at each step of analysis in the generation of the SCN circadian proteome. Left: filtering of identified proteins (white) to quantified proteins (pink). Middle: application of the circadian analysis algorithms RAIN (blue) and JTK-cycle (green) along with the overlap between the two algorithms (teal). Right: final SCN circadian proteome (teal) consisting of 153 proteins.

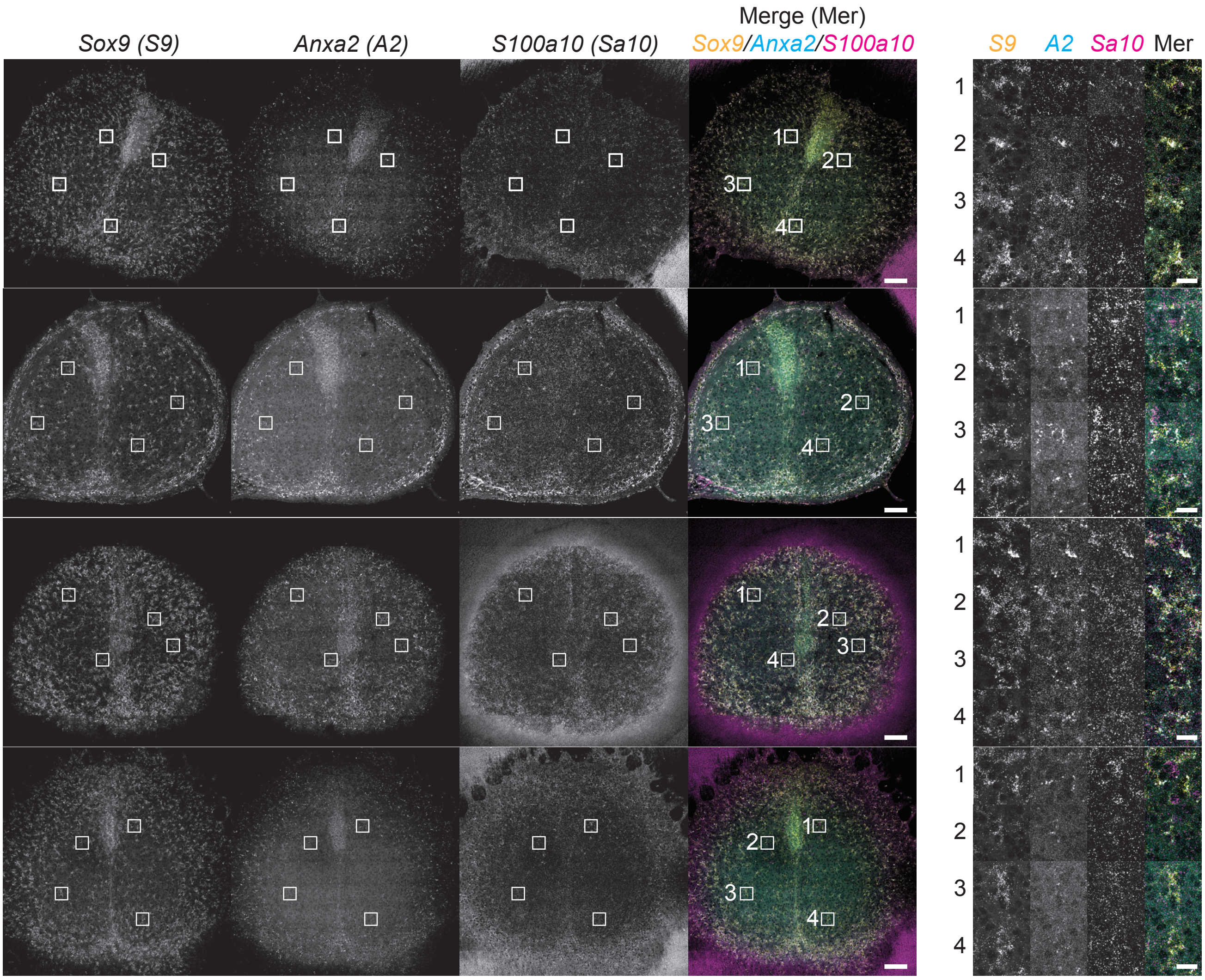

Supplementary Figure 2. Additional fluorescence *in situ* hybridisation images showing *Anxa2*, *S100a10* and *Sox9* co-localisation in SCN explants.

Confocal micrographs of fluorescent *in situ* hybridisation for Sox9 (far left), Anxa2 (middle left) and S100a10 (middle right) in SCN slices alongside a merged image (far right). Insets of the boxed regions in the larger micrographs depicting cells co-expressing the transcripts are shown on the ends of the rows. Each row represents an individual SCN slice (N =4). Scale bars are 100μm/20μm.

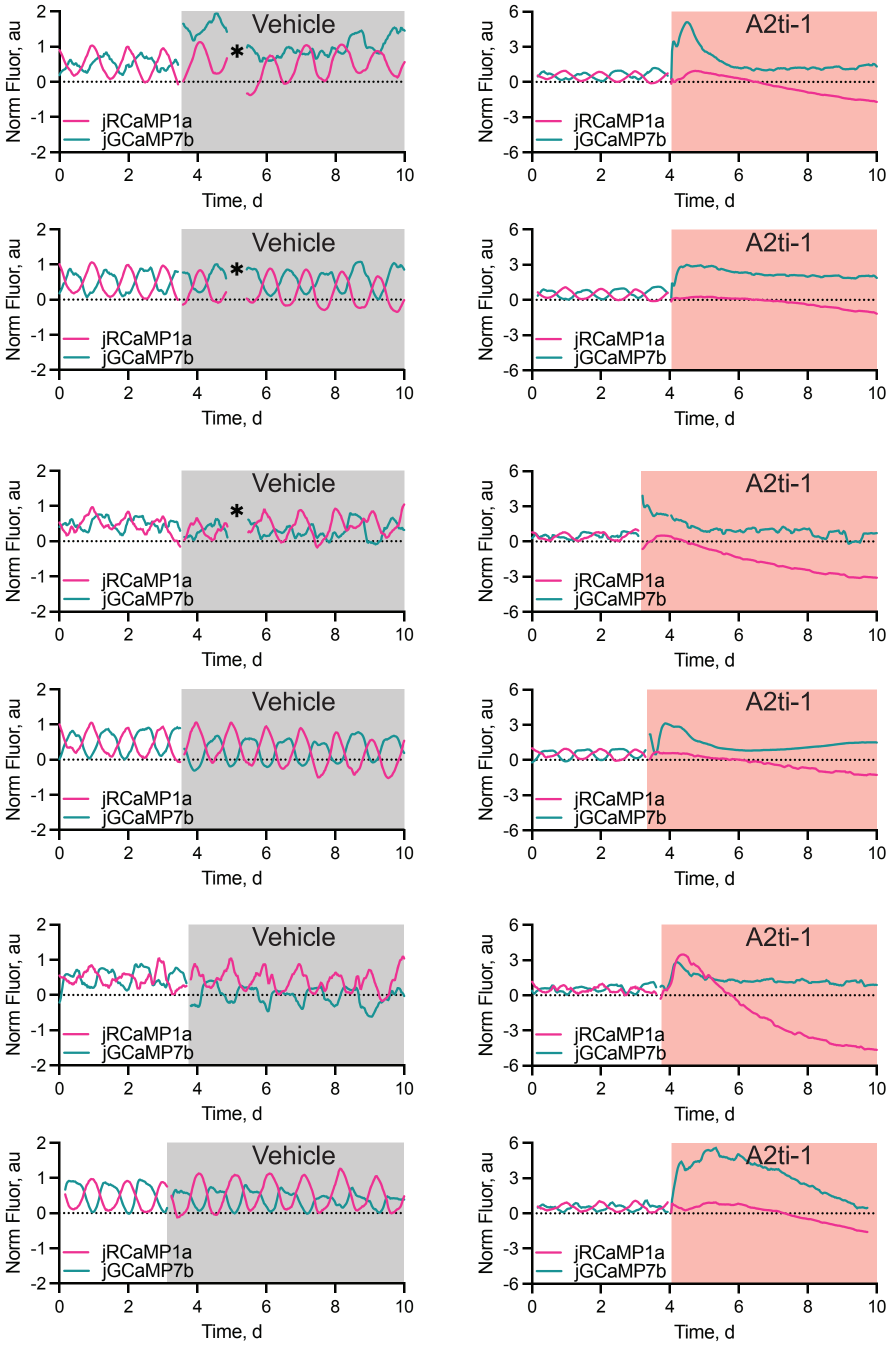

Supplementary Figure 3. Individual multiplexed fluorescence traces for GFAP.jGCaMP7b and Syn.jRCaMP1a from SCN slices treated with vehicle or 80µM A2ti-1.

Normalised, detrended fluorescence for neuronal $[Ca^{2+}]_i$ (jRCaMP1a, pink) and astrocytic $[Ca^{2+}]_i$ (jGCaMP7b, teal) from individual slices treated with vehicle (left, grey shading) or 80µM A2ti-1 (right, pink shading) that are combined as the aggregate mean traces shown in Figure 4E. Slices received both vehicle and A2ti-1 treatment, so paired recordings from the same SCN are shown on the same row. Asterisks indicate missing data due to a system malfunction.

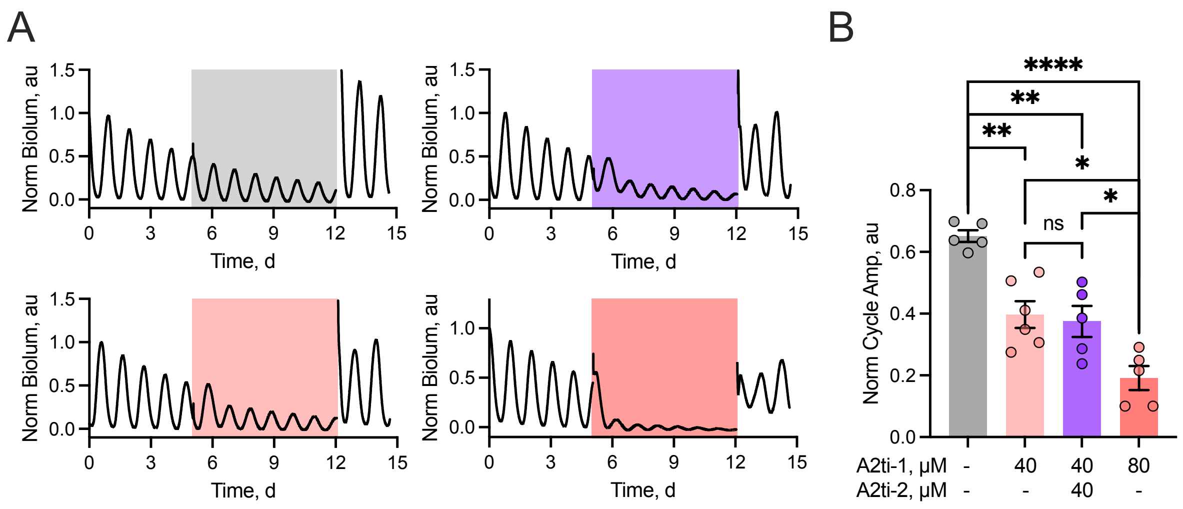

Supplementary Figure 4. A2ti-2 compound does not modify the effects of A2ti-1 treatment.

(A) Normalised Per2::Luc traces showing pre- and post-treatment with vehicle (grey, upper left), 40μM A2ti-1 (light red, lower left), 40μM A2ti-1 and 40μM A2ti-2 (purple, upper right) combined, and 80μM A2ti-1 (red, lower right). Treatment intervals are shown by shading. The final concentration of DMSO (vehicle) in all experiments was 0.2%. (B) Summary histogram showing normalised amplitude for slices treated with different doses of A2ti compounds. Statistics: One-way ANOVA, $F(3, 17) = 20.97$, $p<0.0001$, Post-hoc Šidák's Multiple Comparisons: ****$p<0.0001$, **$p<0.002$, *$p<0.04$, ns $p=0.99$. Group sizes are: 5 Vehicle, 6 40μM A2ti-1, 5 40μM A2ti-1 and 40μM A2ti-2, and 5 80μM A2ti-1 treated SCN explants.

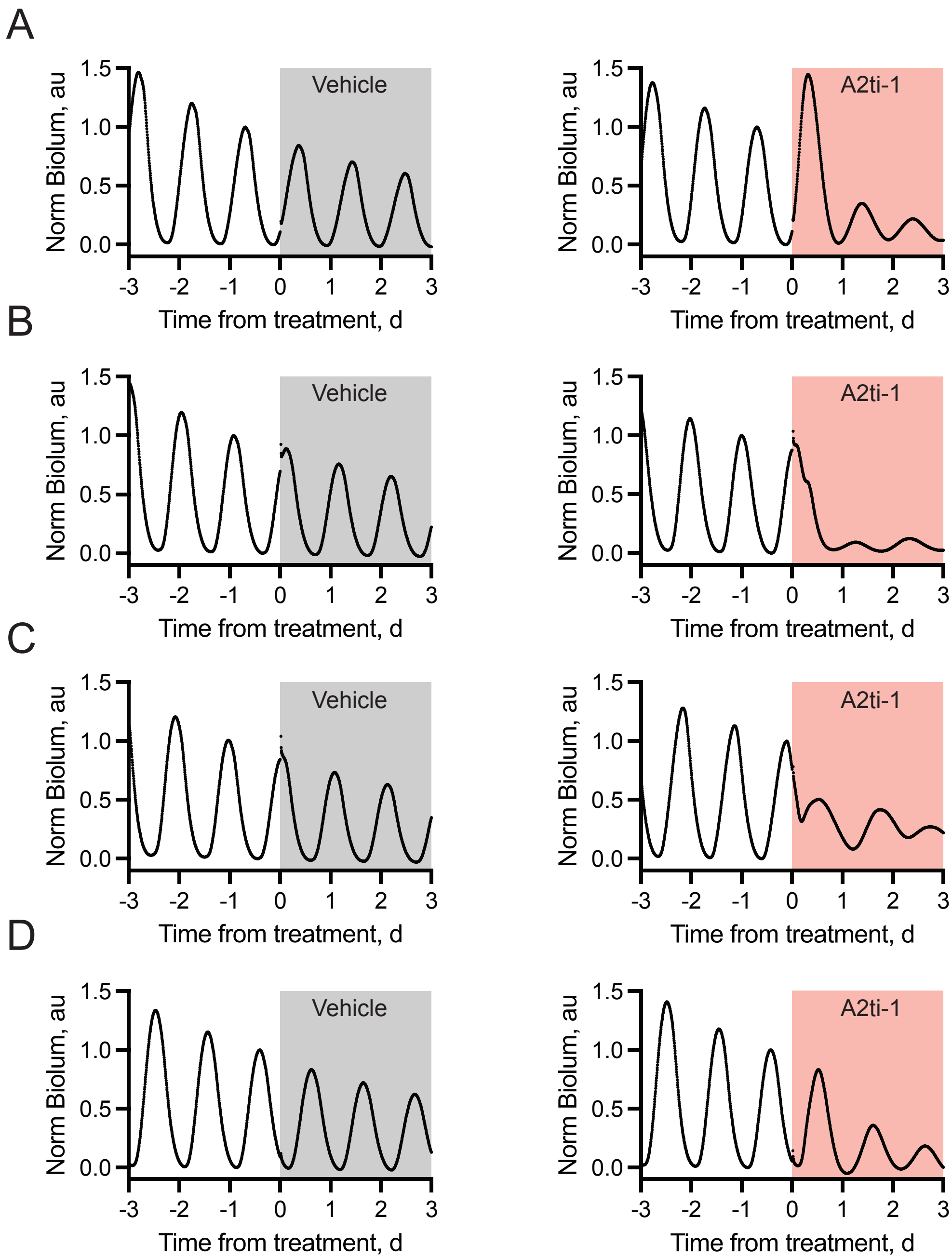

Supplementary Figure 5. Example Per2::Luc bioluminescence traces showing bi-directional phase-specific acute effects of 80μM A2ti-1 treatment.

Normalised Per2::Luc bioluminescence traces showing recordings for 3 days before and 3 days during treatment with vehicle (left, grey) or 80μM A2ti-1 (right, pink), applied at different circadian phases: CT0-6 (A), CT6-12 (B), CT12-18 (C) and CT18-24 (D). Note that the amplitude of the first cycle following treatment for vehicle is stable, whereas the effect of A2ti-1 varies with treatment phase, showing a variety of responses: enhancement (CT0-6, A), suppression (CT6-12, B and CT12-18, C) and no response (CT18-24, D).

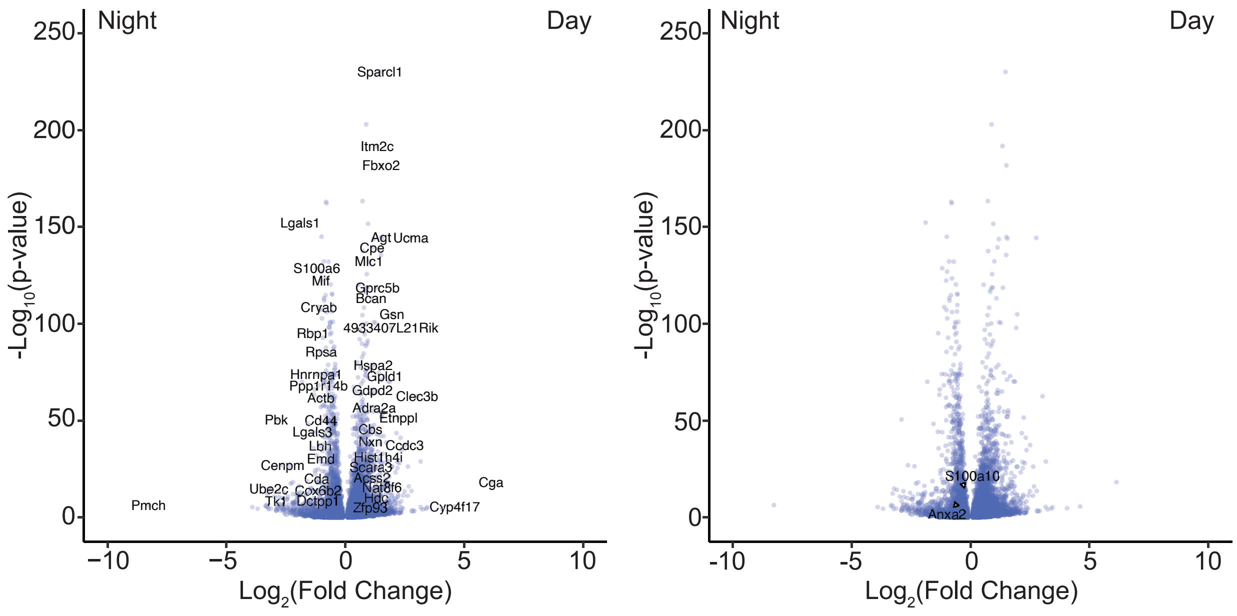

Supplementary Figure 6. Volcano plots showing circadian phase (day vs. night) differences in the expression of *Anxa2* and *S100a10* within the transcriptome of the SCN astrocytes.

Left: comprehensive plot of day-night regulated genes identified by differential expression analysis. Right: the same volcano plot highlighting the relatively weak circadian phase difference for expression levels of *Anxa2* and *S100a10*.

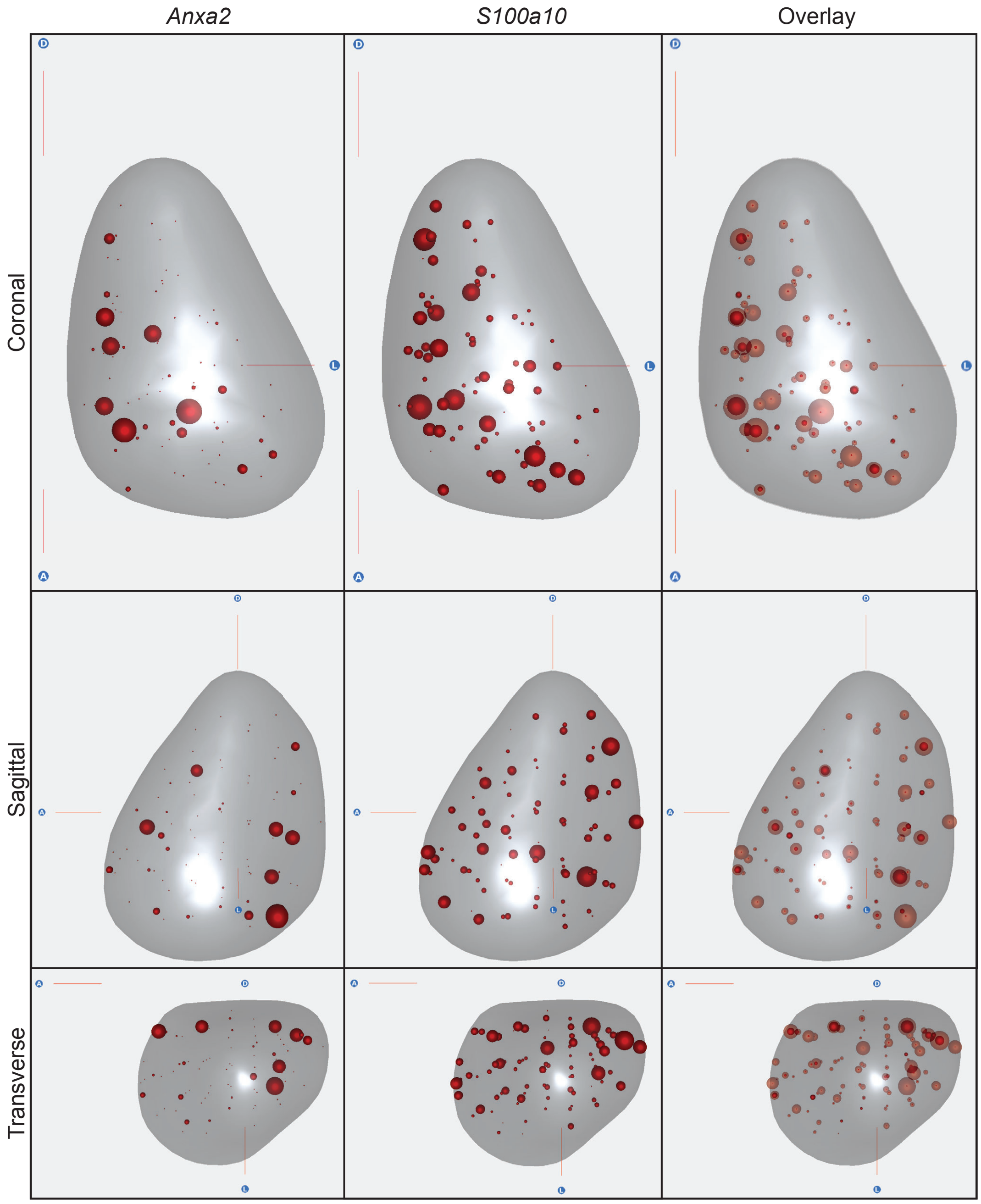

Supplementary Figure 7. Graphical representation of co-expression of *Anxa2* and *S100a10* within discrete regions of the SCN.

3D expression projection map from the Yan Laboratory SCN 3D atlas (Wen et al., 2020) (http://yanlab.org.cn/scn-atlas, accessed 11th October 2024) showing coronal (upper), sagittal (middle) and transverse (lower) views of the SCN model for: *Anxa2* (left), *S100a10* (middle) and an overlay of *Anxa2* and *S100a10* data (where *S100a10* is made transparent) (right). Expression data are from RNA-seq of localised micro-dissections of SCN tissue (Wen et al., 2020). Red lines indicate orientation in 3D-space.

Supplementary R Analysis Scripts

```
#Libraries ----

library(rain)
library(lattice)
library(plyr)
library(Seurat)
library(dplyr)
library(patchwork)
library(hdf5r)
library(ggplot2)
library(tidytable)
library(readxl)
library(forcats)
library(pheatmap)
library(viridis)
library(caret)
library(org.Mm.eg.db)
library(clusterProfiler)
library(AnnotationHub)
library(tidyverse)
library(VennDiagram)
library(DOSE)
library(EnhancedVolcano)
library(nlme)
library(emmeans)

#Environment setup ----

set.seed(42)


#Circadian analysis of Quantified Proteins ----

#JTK cycle ----

source("/JTKversion3/JTK_CYCLEv3.1.R")

project <- "SCNProt.JTK"

options(stringsAsFactors=FALSE)
annot <- read.delim("Annotation_File.txt")

#Proteins column of Quantified Proteome

data <- read.delim("Quantified_Proteome.txt")

rownames(data) <- data[,1]
data <- data[,-1]
jtkdist(5,3)

periods <- 4:4  #Only allow 24h periods
```

```
jtk.init(periods,6)

cat("JTK analysis started on",date(),"\n")
flush.console()

st <- system.time({
  JTK_res <- apply(data,1,function(z) {
    jtkx(z)
    c(JTK.ADJP,JTK.PERIOD,JTK.LAG,JTK.AMP)
  })
  JTK_res <- as.data.frame(t(JTK_res))
  bhq <- p.adjust(unlist(JTK_res[,1]),"BH")
  JTK_res <- cbind(bhq,JTK_res)
  colnames(JTK_res) <- c("BH.Q","ADJ.P","PER","LAG","AMP")
  JTK_res <- cbind(annot,JTK_res,data)
  JTK_res <- JTK_res[order(JTK_res$ADJ.P,-JTK_res$AMP),]
})
print(st)

setwd("/R/Data Output/")

save(JTK_res,file=paste("JTK",project,"rda",sep="."))
write.table(JTK_res,file=paste("JTK",project,"txt",sep="."),
            row.names=F,col.names=T,quote=F,sep="\t")

#RAIN ----

project2<-"SCNProt.RAIN"

Rain_res <- rain(t(data),
                 deltat=6,
                 period=24,
                 nr.series=3,
                 peak.border=c(0.3, 0.7),
                 verbose=FALSE,
                 method="independent")

Names <- rownames(Rain_res)
Rain_res <- cbind(Names, Rain_res)

Rain_res <- arrange(Rain_res, Names)

setwd("/R/Data Output")

write.table(Rain_res,file=paste("Merged",project,"txt",sep="."),
            row.names=F,col.names=T,quote=F,sep="\t")



#Generate genelists & Venn overlap ----

JTK_pval <- filter(JTK_res, ADJ.P < 0.05)
RAIN_pval <- filter(Rain_res, pVal < 0.05)

setwd("/R/Plots")
```

```
venn.diagram(x = list("JTK" = JTK_pval$Probe,
                      "RAIN" = RAIN_pval$Names),
             filename = 'JTK&RAINVENN.tiff')

CP_Overlap <- calculate.overlap(list("JTK" = JTK_pval$Probe,
                                     "RAIN" = RAIN_pval$Names))

CP_List <- as.data.frame(CP_Overlap$a3)
CP_List <- CP_List$`CP_Overlap$a3`

CP_overlap_data <- tibble(JTK_res[c(1,7:21)])
CP_overlap_data <- filter(CP_overlap_data, Probe %in% CP_List)

JTK_table <- tibble(JTK_res[1:6]) %>% filter(Probe %in% CP_List) %>%
arrange(Probe)
RAIN_table <- tibble(Rain_res) %>% filter(Names %in% CP_List) %>%
arrange(Names)
CP_align_table <- tibble(JTK_res[c(1,7:21)]) %>% filter(Probe %in%
CP_List) %>% arrange(Probe)
CP_Overlap_table <- cbind(RAIN_table, JTK_table[2:6],
CP_align_table[2:16]) %>%
  arrange(pVal) %>%
  rename(RAIN_pVal = pVal, RAIN_phase = phase, RAIN_period = period,
JTK_BH.Q = BH.Q, JTK_ADJ.P = ADJ.P, JTK_PER = PER, JTK_LAG = LAG,
JTK_AMP = AMP)

write.csv(CP_Overlap_table, file = "/R/Data Output/CircProt_RAIN-
JTKOUTPUT.csv")


tm <- tibble(CP_overlap_data$Probe)
tm$Probe <- CP_overlap_data$Probe
tm$CT0 <- rowMeans(CP_overlap_data[2:4])
tm$CT6 <- rowMeans(CP_overlap_data[5:7])
tm$CT12 <- rowMeans(CP_overlap_data[8:10])
tm$CT18 <- rowMeans(CP_overlap_data[11:13])
tm$CT24 <- rowMeans(CP_overlap_data[14:16])

tok_mat <- as.matrix(tm[3:7])

rownames(tok_mat) <- tm$Probe

#Heatmap clustering of circadian proteome ----

ph_k <- pheatmap(tok_mat,
               cluster_row = T,
               cluster_col = F,
               treeheight_row = 0,
               kmeans_k = 4,
               scale = 'row',
               color = viridis(n=256))

tok_mat_exp <- as.data.frame(tok_mat)
tok_mat_exp$cluster <- ph_k$kmeans$cluster
```

```
write.csv(tok_mat_exp, file = '/R/Data
Output/JR_Zscore_ClusterList.csv')

#Reorder Zscore matrix in Excel

tok_mat_ordered <- read.csv("/R/Data Output/JR_Zscore_KOrdered.csv",
                   row.names = 1)

ph_ordered <- pheatmap(tok_mat_ordered[1:5],
                 cluster_row = F,
                 cluster_col = F,
                 treeheight_row = 0,
                 scale = 'row',
                 color = viridis(n=256))

labs.row <- rownames(tok_mat_ordered)
skip <- seq(1,nrow(tok_mat_ordered), by = 3)
labs.row[-skip] <- " "

graphics.off()

setwd("/R/Plots")

pheatmap(tok_mat_ordered[1:5],
         cluster_rows = F,
         cluster_cols = F,
         scale = 'row',
         fontsize_row = 8,
         border_color = NA,
         color = viridis(n=256),
         filename = "JR_heatmap.pdf",
         height = 12,
         width = 6)

graphics.off()

#scale row rescales by centering each row around mean and dividing
by its stdev.



#Seurat clustering of scRNASeq data ----

#Import the required data ----

mmSCN5.data <-
Read10X_h5('GSM5115763_mmSCN5_filtered_gene_bc_matrices_h5.h5')
mmSCN10.data <-
Read10X_h5('GSM5115764_mmSCN10_filtered_gene_bc_matrices_h5.h5')
mmSCN6.data <- Read10X_h5('/
GSM5115760_mmSCN6_filtered_gene_bc_matrices_h5.h5')
mmSCN7.data <-
Read10X_h5('GSM5115761_mmSCN7_filtered_gene_bc_matrices_h5.h5')
mmSCN11.data <-
Read10X_h5('GSM5115762_mmSCN11_filtered_gene_bc_matrices_h5.h5')
```

```
#Create Seurat objects from data ----

mmSCN5 <- CreateSeuratObject(counts = mmSCN5.data, project =
"mmSCN5", min.cells = 3, min.features = 200)
mmSCN10 <- CreateSeuratObject(counts = mmSCN10.data, project =
"mmSCN10", min.cells = 3, min.features = 200)
mmSCN6 <- CreateSeuratObject(counts = mmSCN6.data, project =
"mmSCN6", min.cells = 3, min.features = 200)
mmSCN7 <- CreateSeuratObject(counts = mmSCN7.data, project =
"mmSCN7", min.cells = 3, min.features = 200)
mmSCN11 <- CreateSeuratObject(counts = mmSCN11.data, project =
"mmSCN11", min.cells = 3, min.features = 200)

rm(mmSCN5.data, mmSCN10.data, mmSCN6.data, mmSCN7.data,
mmSCN11.data)
gc()

#Merge all datasets ----

All <- merge(mmSCN5, y = c(mmSCN10, mmSCN6, mmSCN7, mmSCN11),
             add.cell.ids = c("SCN5", "SCN10", "SCN6", "SCN7",
"SCN11"), project = "All")
All
rm(mmSCN5, mmSCN6,mmSCN7, mmSCN10, mmSCN11)
gc()

#QC - remove mito genes ----

All[["percent.mt"]] <- PercentageFeatureSet(All, pattern = "^mt-")
VlnPlot(All, features = c("nFeature_RNA", "nCount_RNA",
"percent.mt"), ncol = 3)
All <- subset(All, subset = nFeature_RNA > 1000 & nFeature_RNA <
7000 & percent.mt < 12.5)

#Normalise the data and cluster ----

All <- NormalizeData(All, normalization.method = "LogNormalize",
scale.factor = 10000)
All <- FindVariableFeatures(All, selection.method = "vst", nfeatures
= 2000)
all.genes <- rownames(All)
All <- ScaleData(All, features = all.genes)
All <- JoinLayers(All)
All <- RunPCA(All, features = VariableFeatures(object = All),
npcs=100)

#OPTIONAL: Check the number of dimensions to use from the PCA ----

#ElbowPlot(All, ndims = 50, reduction = "pca")

#From ElbowPlot, looks like 40 represents the elbow (very little
decline after 40)
#Can also use JackStraw here, but probabilities stay significant for
massive number of dimensions
#at this stage, separating the cell-types can see the PCs that
define these at v. low dimensionality
```

```
All <- FindNeighbors(All, dims = 1:40)

#OPTIONAL: Check clustering using clustree ----
#Clustree lets you see what resolution to pass to FindClusters

#resolution.range <- seq(from = 0, to = 2, by = 0.05)
#All <- FindClusters(All, resolution = resolution.range, method =
"igraph", algorithm = 3)
#ClustreePlot <- clustree(All, prefix = 'RNA_snn_res.')

#Use resolution of 1.2 (clusters are reasonably stable at this
point)

#Find Clusters and run/plot UMAP ----

All <- FindClusters(All, resolution = 1.2, method = "igraph",
algorithm = 3)

#Use the iGraph method to avoid sending massive dataset to matrix,
use alogrithm 3 (SLM)

All <- RunUMAP(All, dims = 1:40, random.seed = 42)
DimPlot.NoID <- DimPlot(All, reduction = "umap", label = TRUE)

#Save file (1) ----
saveRDS(All, file = "/R/scData/All.rds")

#Assign cell identities to clusters ----

#Look at top 50 gene lists for each cluster to help assign extra-SCN
and SCN neurons

all_markers <-FindAllMarkers(All, min.pct =  0.25,
                             min.diff.pct = 0.25)

#Create function to obtain top 50 genes

gen_marker_table <- function(x){
  all_markers[all_markers$cluster == x, ] %>%
    head(n=50)
}

#Run function to create gene table

top50_markers <- map_dfr(0:16, gen_marker_table)
View(top50_markers)

#Assign SCN neurons as: Cluster 0-7,9-10,13,16 based on being
GABAergic (Slc32a1+) and
#expression of Nms, Vip, Avp, Penk, Prok2, Grp and/or Chgb.
#Assign Extra-SCN neurons as: Cluster 18, 26 and 27 based on being
Glutamatergic (Slc17a6+) and
#(in the case of GABAergic neuron) expression of Th (Dop+), Sst/Agrp
together and/or expression of Arhgdig (No SCN exp)
```

```
#and Rgs10 (No SCN exp) - (Rgs10 and Arhgdig are expressed in area
around SCN with little SCN exp).
#Assign Astrocytes as: Cluster 8,11 and 24 based on Gfap, Aldh1l1,
Aqp4, Agt and Sox9 expression.
#Assign Ependymocytes as: Cluster 15 based on Tmem212, Tctex1d4
expression.
#Assign Tanycytes as: Cluster 22 based on Col23a1 expression.
#Assign Microglia as: Cluster 17 and 20 based on Ly86, C1qa, Hexb
expression.
#Assign NG2 cells as: Cluster 12 based on Pdgfra expression.
#Assign Radial Glia as: Cluster 19 based on Ccnb1, Ube2c expression.
#Assign Oligodendrocytes as: Cluster 14 based on Mog, Plp1
expression.
#Assign Endothelial cells as: Cluster 21 and 25 based on Lum, Dcn
expression.
#Assign Unclassified cells as: Cluster 23, 28 and 29 based on
exclusion of other markers.

#Rename clusters to regroup ----

All.named <- All

clusterIDs <- read_xlsx('/R/scData/Cluster Identities.xlsx')
cluster_names <- clusterIDs$Name
names(cluster_names) <- levels(All.named)

All.named <- RenameIdents(All.named, cluster_names)
saveRDS(All.named, file = "/R/scData/All.named.rds")

All.named.DimPlot <- DimPlot(All.named, reduction = "umap", label =
TRUE)

All.named.DimPlot

#Create summary table of numbers of cells in each group (and %) ----

CellsID <- summary(All.named@active.ident)
CellsID <- as_tibble(CellsID, rownames = "CellType")
CellsID$CellType <- as.factor(CellsID$CellType)
CellsID <- rename(CellsID, "Counts" = "value")
CellsID$Percent <- (CellsID$Counts/sum(CellsID$Counts))*100
View(CellsID)

#Create heatmap for top 5 upregulated genes in named clusters ----

TopClusterGenes <- FindAllMarkers(All.named,
                                  test.use = "wilcox",
                                  random.seed = 42,
                                  only.pos = TRUE)

write.csv(TopClusterGenes,
          row.names = TRUE,
          file = '/R/Data Output/TopUpregClusterGenes.csv')

top5_marker_table <- function(x){
  TopClusterGenes[TopClusterGenes$cluster == x, ] %>%
```

```
    head(n=5)
}

clusters <- CellsID$CellType

top5_markers_named <- map_dfr(clusters, top5_marker_table)

ave_exp_named <- AverageExpression(All.named, features =
top5_markers_named$gene)

All.named.heatmap <- pheatmap(ave_exp_named$RNA,
                              cluster_rows = FALSE, cluster_cols =
FALSE,
                              scale = "row", border_color = "white",
                              color = viridis(n = 256, alpha = 1,
begin = 0, end = 1,
                                              option = "viridis"))

All.named.heatmap

#To get a correctly ordered heatmap that is aesthetically pleasing:

top5_markers_named <- map_dfr(clusters, top5_marker_table)

order <- c(rep(1,5),
           rep(3,5),
           rep(6,5),
           rep(5,5),
           rep(4,5),
           rep(9,5),
           rep(2,5),
           rep(7,5),
           rep(10,5),
           rep(8,5),
           rep(11,5))

top5_markers_named$order <- order
top5_markers_named <-
top5_markers_named[order(top5_markers_named$order),]

ave_exp_named <- AverageExpression(All.named, features =
top5_markers_named$gene)

col_order <- c("SCN Neurons",
               "Extra-SCN Neurons",
               "Astrocytes",
               "Ependymocytes",
               "Oligodendrocytes",
               "NG2 Cells",
               "Radial Glia",
               "Tanycytes",
               "Microglia",
               "Endothelial Cells",
               "Unclassified")

row_order <- top5_markers_named$gene
```

```
ave_exp_named$RNA <- ave_exp_named$RNA[row_order,col_order]
breakslimit = seq(0,1, by = 1/256)

All.named.heatmap <- pheatmap(ave_exp_named$RNA,
                              cluster_rows = FALSE, cluster_cols =
FALSE,
                              scale = "row", border_color = "white",
                              color = viridis(n = 256, alpha = 1,
begin = 0, end = 1,
                                              option = "viridis"),
breaks = breakslimit,
                              filename = "Cluster_heatmap.pdf",
                              height = 10.65,
                              width = 6.36)

All.named.heatmap

#GO analysis ---

#Find all markers

all_markers_named_pos <- FindAllMarkers(All.named,
                                    test.use = "wilcox",
                                    min.pct =  0.25,
                                    min.diff.pct = 0.25,
                                    random.seed = 42,
                                    only.pos = T)

#Generate marker table function ----

gen_marker_table <- function(x){
  all_markers_named_pos[all_markers_named_pos$cluster == x, ] %>%
    head(n=250)
}

#Generate table of top150 upregulated genes for each cluster ----

clusternames <- levels(all_markers_named_pos$cluster)

all_markers_named_pos <- filter(all_markers_named_pos, p_val_adj <
0.05)

all_markers_named_pos <-
all_markers_named_pos[order(all_markers_named_pos$p_val_adj),]

top250_pos <- map_dfr(clusternames, gen_marker_table)
View(top250_pos)

#Create dataframe to get GO terms from clusterprofiler ----

df <- top250_pos[,7:6]
dfsample <- split(df$gene,df$cluster)
length(dfsample)
```

```
dfsample

dfsample$`SCN Neurons` = bitr(dfsample$`SCN Neurons`,
fromType="SYMBOL", toType="ENTREZID", OrgDb="org.Mm.eg.db")
dfsample$`Extra-SCN Neurons` = bitr(dfsample$`Extra-SCN Neurons`,
fromType="SYMBOL", toType="ENTREZID", OrgDb="org.Mm.eg.db")
dfsample$`Astrocytes` = bitr(dfsample$`Astrocytes`,
fromType="SYMBOL", toType="ENTREZID", OrgDb="org.Mm.eg.db")
dfsample$`NG2 Cells` = bitr(dfsample$`NG2 Cells`, fromType="SYMBOL",
toType="ENTREZID", OrgDb="org.Mm.eg.db")
dfsample$`Oligodendrocytes` = bitr(dfsample$`Oligodendrocytes`,
fromType="SYMBOL", toType="ENTREZID", OrgDb="org.Mm.eg.db")
dfsample$`Ependymocytes` = bitr(dfsample$`Ependymocytes`,
fromType="SYMBOL", toType="ENTREZID", OrgDb="org.Mm.eg.db")
dfsample$`Microglia` = bitr(dfsample$`Microglia`, fromType="SYMBOL",
toType="ENTREZID", OrgDb="org.Mm.eg.db")
dfsample$`Radial Glia` = bitr(dfsample$`Radial Glia`,
fromType="SYMBOL", toType="ENTREZID", OrgDb="org.Mm.eg.db")
dfsample$`Tanycytes` = bitr(dfsample$`Tanycytes`, fromType="SYMBOL",
toType="ENTREZID", OrgDb="org.Mm.eg.db")
dfsample$`Endothelial Cells` = bitr(dfsample$`Endothelial Cells`,
fromType="SYMBOL", toType="ENTREZID", OrgDb="org.Mm.eg.db")
dfsample$`Unclassified` = bitr(dfsample$`Unclassified`,
fromType="SYMBOL", toType="ENTREZID", OrgDb="org.Mm.eg.db")

genelist <- list("SCN Neurons" = dfsample$`SCN Neurons`$ENTREZID,
                 "Extra-SCN Neurons" = dfsample$`Extra-SCN
Neurons`$ENTREZID,
                 "Astrocytes" = dfsample$`Astrocytes`$ENTREZID,
                 "Ependymocytes" =
dfsample$`Ependymocytes`$ENTREZID,
                 "Oligodendrocytes" =
dfsample$`Oligodendrocytes`$ENTREZID,
                 "NG2 Cells" = dfsample$`NG2 Cells`$ENTREZID,
                 "Radial Glia" = dfsample$`Radial Glia`$ENTREZID,
                 "Tanycytes" = dfsample$`Tanycytes`$ENTREZID,
                 "Microglia" = dfsample$`Microglia`$ENTREZID,
                 "Endothelial Cells" = dfsample$`Endothelial
Cells`$ENTREZID,
                 "Unclassified" = dfsample$`Unclassified`$ENTREZID)

#Run clusterprofiler ----

GOclusterplot <- compareCluster(geneCluster = genelist,
                                fun = "enrichGO",
                                ont = "MF",
                                OrgDb = "org.Mm.eg.db")

dotclusterplot <- dotplot(GOclusterplot,
                          showCategory = 2,
                          color = "p.adjust",
                          x = "Cluster",
                          font.size = 10) +
  scale_fill_viridis(direction = -1)

ggsave(filename = "GO_Clusterplot.pdf",
```

```
       plot = dotclusterplot,
       device = "pdf",
       height = 8,
       width = 6,
       units= "in",
       dpi = 600)

#Generate results tables ----

GOResults <- GOclusterplot@compareClusterResult

GOResults$Gene.Ratio <- parse_ratio(GOResults$GeneRatio)
GOResults$Bg.Ratio <- parse_ratio(GOResults$BgRatio)
GOResults$FoldEnrich <-
((GOResults$Gene.Ratio*100)/(GOResults$Bg.Ratio*100))

#Generate gene lists to obtain GO terms using enrichGO

Astro_RNA_table <- filter(all_markers_named_pos, cluster ==
"Astrocytes")
Astro_RNA <- Astro_RNA_table$gene

venn.input <- list("RNA" = Astro_RNA, "Protein" = CP_List)

RNA_Prot_overlap <- calculate.overlap(venn.input)
RNA_Prot_Venn <- venn.diagram(venn.input, filename =
'RNA_Prot_Venn.tiff')

RNA_Prot_Intersect <- RNA_Prot_overlap$a3

#Get GO term enrichment using enrichGO ----

GO_Circ_proteome <- enrichGO(CP_List,
                             org.Mm.eg.db,
                             keyType = "SYMBOL",
                             ont = "MF",
                             pAdjustMethod = "BH",
                             pvalueCutoff = 0.05)

GO_Circ_proteome_Res <- GO_Circ_proteome@result
GO_Circ_proteome_Res$Gene.Ratio <-
parse_ratio(GO_Circ_proteome_Res$GeneRatio)
GO_Circ_proteome_Res$Bg.Ratio <-
parse_ratio(GO_Circ_proteome_Res$BgRatio)
GO_Circ_proteome_Res$FoldEnrich <-
((GO_Circ_proteome_Res$Gene.Ratio*100)/(GO_Circ_proteome_Res$Bg.Rati
o*100))

GO_Astro_RNA <- enrichGO(Astro_RNA,
                         org.Mm.eg.db,
                         keyType = "SYMBOL",
                         ont = "MF",
                         pAdjustMethod = "BH",
                         pvalueCutoff = 0.05)

GO_Astro_RNA_Res <- GO_Astro_RNA@result
```

```
GO_Astro_RNA_Res$Gene.Ratio <-
parse_ratio(GO_Astro_RNA_Res$GeneRatio)
GO_Astro_RNA_Res$Bg.Ratio <- parse_ratio(GO_Astro_RNA_Res$BgRatio)
GO_Astro_RNA_Res$FoldEnrich <-
((GO_Astro_RNA_Res$Gene.Ratio*100)/(GO_Astro_RNA_Res$Bg.Ratio*100))


GO_RNA_Prot_Intersect <- enrichGO(RNA_Prot_Intersect,
                                  org.Mm.eg.db,
                                  keyType = "SYMBOL",
                                  ont = "MF",
                                  pAdjustMethod = "BH",
                                  pvalueCutoff = 0.05)

GO_RNA_Prot_Intersect_Res <- GO_RNA_Prot_Intersect@result
GO_RNA_Prot_Intersect_Res$Gene.Ratio <-
parse_ratio(GO_RNA_Prot_Intersect_Res$GeneRatio)
GO_RNA_Prot_Intersect_Res$Bg.Ratio <-
parse_ratio(GO_RNA_Prot_Intersect_Res$BgRatio)
GO_RNA_Prot_Intersect_Res$FoldEnrich <-
((GO_RNA_Prot_Intersect_Res$Gene.Ratio*100)/(GO_RNA_Prot_Intersect_R
es$Bg.Ratio*100))

setwd("/R/Data Output/")

write.csv(GO_Circ_proteome_Res, file = "CircProt_GOTerms.csv")
write.csv(GOResults, file = "RNA-Cluster_GOTerms.csv")
write.csv(GO_Astro_RNA, file = "Astro_RNA_GOTerms.csv")
write.csv(GO_RNA_Prot_Intersect_Res, file = "RNA-
Prot_Intersect_GOTerms.csv")

#Cnet plots showing genes associated to GO terms ----

#Colour maps for category nodes to match up to edge colours

colour.map.CircProt <- c("#DB8E00",
                         "#F8766D",
                         "#64B200",
                         "#AEA200",
                         "#EF67EB",
                         "#00BD5C",
                         "#00C1A7",
                         "#B385FF",
                         "#FF63B6",
                         "#00A6FF")

colour.map.RNAAstro <- c("#64B200",
                         "#F8766D",
                         "#00BD5C",
                         "#00C1A7",
                         "#B385FF",
                         "#DB8E00",
                         "#AEA200",
                         "#00BADE",
                         "#EF67EB",
                         "#FF63B6")
```

```
colour.map.RPIntersect <- c("#F8766D", #Actin binding 1
                           "#DB8E00", #Actin Filament binding 2
                           "#EF67EB", #S100 3
                           "#64B200", #Ca2+ dep protein binding 4
                           "#FF63B6", #transmembrane 5
                           "#AEA200", #Ca2+ dep phospholipid
binding 6
                           "#B385FF", #Phosphatidyl serine 7
                           "#00BD5C", #MODIFIED AA BINDING 8
                           "#00C1A7", #metal ion transmembrane
trans 9
                           "#00BADE") #PI4,5 bisphosphate binding
10

#Category network plots

cnet_Circ_proteome <- cnetplot(GO_Circ_proteome,
                              showCategory = 10,
                              layout = 'fr',
                              color.params = list(edge = T,
                                                  category =
colour.map.CircProt),
                              cex.params = list(gene_node = 0.1,
                                                gene_label = 0.5))

cnet_Circ_proteome_ul <- cnetplot(GO_Circ_proteome,
                              showCategory = 10,
                              layout = 'fr',
                              color.params = list(edge = T,
                                                  category =
colour.map.CircProt),
                              cex.params = list(gene_node = 0.1,
                                                gene_label = 0.5),
                              node_label = 'gene')

cnet_Astro_RNA <- cnetplot(GO_Astro_RNA,
                          showCategory = 10,
                          layout = 'fr',
                          color.params = list(edge = T,
                                              category =
colour.map.RNAAstro),
                          cex.params = list(gene_node = 0.1,
                                            gene_label = 0.5))

cnet_Astro_RNA_ul <- cnetplot(GO_Astro_RNA,
                          showCategory = 10,
                          layout = 'kk',
                          color.params = list(edge = T,
                                              category =
colour.map.RNAAstro),
                          cex.params = list(gene_node = 0.1,
                                            gene_label = 0.5),
                          node_label = 'gene') +
  guides(edge_color = "none")
```

```
cnet_RP_Intersect <- cnetplot(GO_RNA_Prot_Intersect,
                              showCategory = 10,
                              layout = 'fr',
                              color.params = list(edge = T,
                                                  category =
colour.map.RPIntersect),
                              cex.params = list(gene_node = 0.1,
                                                gene_label = 0.5))

cnet_RP_Intersect_ul <- cnetplot(GO_RNA_Prot_Intersect,
                              showCategory = 10,
                              layout = 'fr',
                              color.params = list(edge = T,
                                                  category =
colour.map.RPIntersect),
                              cex.params = list(gene_node = 0.1,
                                                gene_label = 0.5),
                              node_label = 'gene')

ggsave(filename = "cnet_circprot.tiff",
       plot = cnet_Circ_proteome_ul,
       device = "tiff",
       height = 5,
       width = 7.5,
       units= "in",
       dpi = 600)

ggsave(filename = "cnet_astroRNA.tiff",
       plot = cnet_Astro_RNA_ul,
       device = "tiff",
       height = 10,
       width = 8,
       units= "in",
       dpi = 600)

ggsave(filename = "cnet_RP_Intersect.tiff",
       plot = cnet_RP_Intersect_ul,
       device = "tiff",
       height = 5,
       width = 7.5,
       units= "in",
       dpi = 600)

#Anxa2 and interaction partners heatmap across cells ----

Anxa2Int <- read.csv(file = 'Anxa2_Int.csv',
                     header = FALSE)
Anxa2Int <- Anxa2Int$V1

Anxa2_Int_Data <- FetchData(All.named, vars = c(Anxa2Int,'ident'),
layer = "counts")

#Note: 1 of these genes weren't found in the scRNAseq dataset: Cfd

SCNNeurons_A2 <- Anxa2_Int_Data %>% filter(ident == "SCN Neurons")
%>% select(!ident) %>% plyr::ldply(sum)
```

```
SCNNeurons_A2 <- t(as.data.frame(SCNNeurons_A2$V1, row.names =
SCNNeurons_A2$.id))

ExSCNNeurons_A2 <- Anxa2_Int_Data %>% filter(ident == "Extra-SCN
Neurons") %>% select(!ident) %>% plyr::ldply(sum)
ExSCNNeurons_A2 <- t(as.data.frame(ExSCNNeurons_A2$V1, row.names =
ExSCNNeurons_A2$.id))

Astrocytes_A2 <- Anxa2_Int_Data %>% filter(ident == "Astrocytes")
%>% select(!ident) %>% plyr::ldply(sum)
Astrocytes_A2 <- t(as.data.frame(Astrocytes_A2$V1, row.names =
Astrocytes_A2$.id))

Ependy_A2 <- Anxa2_Int_Data %>% filter(ident == "Ependymocytes") %>%
select(!ident) %>% plyr::ldply(sum)
Ependy_A2 <- t(as.data.frame(Ependy_A2$V1, row.names =
Ependy_A2$.id))

Oligo_A2 <- Anxa2_Int_Data %>% filter(ident == "Oligodendrocytes")
%>% select(!ident) %>% plyr::ldply(sum)
Oligo_A2 <- t(as.data.frame(Oligo_A2$V1, row.names = Oligo_A2$.id))

NG2_A2 <- Anxa2_Int_Data %>% filter(ident == "NG2 Cells") %>%
select(!ident) %>% plyr::ldply(sum)
NG2_A2 <- t(as.data.frame(NG2_A2$V1, row.names = NG2_A2$.id))

RG_A2 <- Anxa2_Int_Data %>% filter(ident == "Radial Glia") %>%
select(!ident) %>% plyr::ldply(sum)
RG_A2 <- t(as.data.frame(RG_A2$V1, row.names = RG_A2$.id))

Tany_A2 <- Anxa2_Int_Data %>% filter(ident == "Tanycytes") %>%
select(!ident) %>% plyr::ldply(sum)
Tany_A2 <- t(as.data.frame(Tany_A2$V1, row.names = Tany_A2$.id))

Micro_A2 <- Anxa2_Int_Data %>% filter(ident == "Microglia") %>%
select(!ident) %>% plyr::ldply(sum)
Micro_A2 <- t(as.data.frame(Micro_A2$V1, row.names = Micro_A2$.id))

Endo_A2 <- Anxa2_Int_Data %>% filter(ident == "Endothelial Cells")
%>% select(!ident) %>% plyr::ldply(sum)
Endo_A2 <- t(as.data.frame(Endo_A2$V1, row.names = Endo_A2$.id))

Unclass_A2 <- Anxa2_Int_Data %>% filter(ident == "Unclassified") %>%
select(!ident) %>% plyr::ldply(sum)
Unclass_A2 <- t(as.data.frame(Unclass_A2$V1, row.names =
Unclass_A2$.id))

ExpMat_A2 <- rbind(SCNNeurons_A2,
                   ExSCNNeurons_A2,
                   Astrocytes_A2,
                   Ependy_A2,
                   Oligo_A2,
                   NG2_A2,
                   RG_A2,
                   Tany_A2,
                   Micro_A2,
```

```
                    Endo_A2,
                    Unclass_A2)

row.names(ExpMat_A2) <- c("SCN Neurons",
                           "Extra-SCN Neurons",
                           "Astrocytes",
                           "Ependymocytes",
                           "Oligodendrocytes",
                           "NG2 Cells",
                           "Radial Glia",
                           "Tanycytes",
                           "Microglia",
                           "Endothelial Cells",
                           "Unclassified")

process <- preProcess(ExpMat_A2, method = "range")
NormExpMat_A2 <- predict(process, ExpMat_A2)

setwd('/R/Plots')

Raw_heatmap_A2 <- pheatmap(ExpMat_A2,
                            cluster_rows = F,
                            cluster_cols = F,
                            color = viridis(n=256, begin = 0, end =
1),
                            border_color = 'white',
                            cellwidth = 10,
                            cellheight = 24,
                            scale = "column")


Norm_heatmap_A2 <- pheatmap(NormExpMat_A2,
                             cluster_rows = F,
                             cluster_cols = F,
                             color = viridis(n=256, begin = 0, end =
1),
                             border_color = 'white',
                             cellwidth = 10,
                             cellheight = 24,
                             filename = "A2_heatmap.pdf",
                             height = 5.3,
                             width = 7.8)

##Reorder heatmap by Anxa2 expression

NEMA2_A2Ordered <- as.data.frame(NormExpMat_A2)
NEMA2_A2Ordered <- NEMA2[order(NEMA2$Anxa2, decreasing = TRUE),]
NEMA2_A2Ordered <- as.matrix(NEMA2_A2Ordered)

Norm_heatmap_A2 <- pheatmap(NEMA2_A2Ordered,
                             cluster_rows = F,
                             cluster_cols = F,
                             color = viridis(n=256, begin = 0, end =
1),
                             border_color = 'white',
                             cellwidth = 10,
```

```
                              cellheight = 24,
                              filename = "A2_ordered_heatmap.pdf",
                              height = 5.3,
                              width = 7.8)

#Expression of S100 proteins proportion expressing both S100a10 and
Anxa2 ----

#Add the time of day information for the harvesting of slices to
metadata ----

All.named$times <- All.named$orig.ident
All.named$times <- fct_collapse(All.named$times,
                                Night = c("mmSCN5", "mmSCN10"),
                                Day = c("mmSCN6", "mmSCN7",
"mmSCN11"))

saveRDS(All.named, file = "/R/scData/All.named.rds")


#Extract normalised expression data per cell for S100a10 and Anxa2 -
--

S100.Anx.Data.All <- FetchData(All.named, vars = c("S100a10",
'Anxa2', 'ident', 'times'))
write.csv(S100.Anx.Data.All,
          row.names = TRUE,
          file = "/R/Data Outputs/S_A_Data_All.csv")

#Plot extracted data for All showing cell-types

#Generate table with % cells expressing different combinations of
S100a10/Anxa2 ----

S100combs <- c("S100a10 & Anxa2", "S100a10 only", "Anxa2 only", "No
S100a10 or Anxa2")

#All cells

S100.Anx.Counts.All <- c(nrow(filter(S100.Anx.Data.All, S100a10 > 0
& Anxa2 > 0)),
                         nrow(filter(S100.Anx.Data.All, S100a10 > 0
& Anxa2 == 0)),
                         nrow(filter(S100.Anx.Data.All, S100a10 == 0
& Anxa2 > 0)),
                         nrow(filter(S100.Anx.Data.All, S100a10 == 0
& Anxa2 == 0)))

S100.Anx.Counts.All.1 <- as_tibble(S100.Anx.Counts.All)

S100.Anx.Counts.All.1$percent <-
((S100.Anx.Counts.All.1$value/sum(S100.Anx.Counts.All.1$value))*100)
S100.Anx.Counts.All.1$combo <- S100combs

#Split by cell types ----
```

```
S100.Anx.Counts.Neurons <- c(nrow(filter(S100.Anx.Data.All, S100a10
> 0 & Anxa2 > 0 & ident == 'SCN Neurons')),
                             nrow(filter(S100.Anx.Data.All, S100a10
> 0 & Anxa2 == 0 & ident == 'SCN Neurons')),
                             nrow(filter(S100.Anx.Data.All, S100a10
== 0 & Anxa2 > 0 & ident == 'SCN Neurons')),
                             nrow(filter(S100.Anx.Data.All, S100a10
== 0 & Anxa2 == 0 & ident == 'SCN Neurons')))

S100.Anx.Counts.ExNeurons <- c(nrow(filter(S100.Anx.Data.All,
S100a10 > 0 & Anxa2 > 0 & ident == 'Extra-SCN Neurons')),
                               nrow(filter(S100.Anx.Data.All,
S100a10 > 0 & Anxa2 == 0 & ident == 'Extra-SCN Neurons')),
                               nrow(filter(S100.Anx.Data.All,
S100a10 == 0 & Anxa2 > 0 & ident == 'Extra-SCN Neurons')),
                               nrow(filter(S100.Anx.Data.All,
S100a10 == 0 & Anxa2 == 0 & ident == 'Extra-SCN Neurons')))

S100.Anx.Counts.Astros <- c(nrow(filter(S100.Anx.Data.All, S100a10 >
0 & Anxa2 > 0 & ident == 'Astrocytes')),
                            nrow(filter(S100.Anx.Data.All, S100a10 >
0 & Anxa2 == 0 & ident == 'Astrocytes')),
                            nrow(filter(S100.Anx.Data.All, S100a10
== 0 & Anxa2 > 0 & ident == 'Astrocytes')),
                            nrow(filter(S100.Anx.Data.All, S100a10
== 0 & Anxa2 == 0 & ident == 'Astrocytes')))

S100.Anx.Counts.Ependy <- c(nrow(filter(S100.Anx.Data.All, S100a10 >
0 & Anxa2 > 0 & ident == 'Ependymocytes')),
                            nrow(filter(S100.Anx.Data.All, S100a10 >
0 & Anxa2 == 0 & ident == 'Ependymocytes')),
                            nrow(filter(S100.Anx.Data.All, S100a10
== 0 & Anxa2 > 0 & ident == 'Ependymocytes')),
                            nrow(filter(S100.Anx.Data.All, S100a10
== 0 & Anxa2 == 0 & ident == 'Ependymocytes')))

S100.Anx.Counts.Oligo <- c(nrow(filter(S100.Anx.Data.All, S100a10 >
0 & Anxa2 > 0 & ident == 'Oligodendrocytes')),
                           nrow(filter(S100.Anx.Data.All, S100a10 >
0 & Anxa2 == 0 & ident == 'Oligodendrocytes')),
                           nrow(filter(S100.Anx.Data.All, S100a10 ==
0 & Anxa2 > 0 & ident == 'Oligodendrocytes')),
                           nrow(filter(S100.Anx.Data.All, S100a10 ==
0 & Anxa2 == 0 & ident == 'Oligodendrocytes')))

S100.Anx.Counts.NG2 <- c(nrow(filter(S100.Anx.Data.All, S100a10 > 0
& Anxa2 > 0 & ident == 'NG2 Cells')),
                         nrow(filter(S100.Anx.Data.All, S100a10 > 0
& Anxa2 == 0 & ident == 'NG2 Cells')),
                         nrow(filter(S100.Anx.Data.All, S100a10 == 0
& Anxa2 > 0 & ident == 'NG2 Cells')),
                         nrow(filter(S100.Anx.Data.All, S100a10 == 0
& Anxa2 == 0 & ident == 'NG2 Cells')))

S100.Anx.Counts.RG <- c(nrow(filter(S100.Anx.Data.All, S100a10 > 0 &
Anxa2 > 0 & ident == 'Radial Glia')),
```

```
                        nrow(filter(S100.Anx.Data.All, S100a10 > 0 &
Anxa2 == 0 & ident == 'Radial Glia')),
                        nrow(filter(S100.Anx.Data.All, S100a10 == 0
& Anxa2 > 0 & ident == 'Radial Glia')),
                        nrow(filter(S100.Anx.Data.All, S100a10 == 0
& Anxa2 == 0 & ident == 'Radial Glia')))

S100.Anx.Counts.Tany <- c(nrow(filter(S100.Anx.Data.All, S100a10 > 0
& Anxa2 > 0 & ident == 'Tanycytes')),
                          nrow(filter(S100.Anx.Data.All, S100a10 > 0
& Anxa2 == 0 & ident == 'Tanycytes')),
                          nrow(filter(S100.Anx.Data.All, S100a10 ==
0 & Anxa2 > 0 & ident == 'Tanycytes')),
                          nrow(filter(S100.Anx.Data.All, S100a10 ==
0 & Anxa2 == 0 & ident == 'Tanycytes')))

S100.Anx.Counts.MG <- c(nrow(filter(S100.Anx.Data.All, S100a10 > 0 &
Anxa2 > 0 & ident == 'Microglia')),
                        nrow(filter(S100.Anx.Data.All, S100a10 > 0 &
Anxa2 == 0 & ident == 'Microglia')),
                        nrow(filter(S100.Anx.Data.All, S100a10 == 0
& Anxa2 > 0 & ident == 'Microglia')),
                        nrow(filter(S100.Anx.Data.All, S100a10 == 0
& Anxa2 == 0 & ident == 'Microglia')))

S100.Anx.Counts.Endo <- c(nrow(filter(S100.Anx.Data.All, S100a10 > 0
& Anxa2 > 0 & ident == 'Endothelial Cells')),
                          nrow(filter(S100.Anx.Data.All, S100a10 > 0
& Anxa2 == 0 & ident == 'Endothelial Cells')),
                          nrow(filter(S100.Anx.Data.All, S100a10 ==
0 & Anxa2 > 0 & ident == 'Endothelial Cells')),
                          nrow(filter(S100.Anx.Data.All, S100a10 ==
0 & Anxa2 == 0 & ident == 'Endothelial Cells')))

S100.Anx.Counts.Unc <- c(nrow(filter(S100.Anx.Data.All, S100a10 > 0
& Anxa2 > 0 & ident == 'Unclassified')),
                         nrow(filter(S100.Anx.Data.All, S100a10 > 0
& Anxa2 == 0 & ident == 'Unclassified')),
                         nrow(filter(S100.Anx.Data.All, S100a10 == 0
& Anxa2 > 0 & ident == 'Unclassified')),
                         nrow(filter(S100.Anx.Data.All, S100a10 == 0
& Anxa2 == 0 & ident == 'Unclassified')))

S100.Anx.CellTypes <- cbind(S100.Anx.Counts.All,
S100.Anx.Counts.Neurons,
                            S100.Anx.Counts.ExNeurons,
S100.Anx.Counts.Astros,
                            S100.Anx.Counts.Ependy,
S100.Anx.Counts.Oligo,
                            S100.Anx.Counts.NG2, S100.Anx.Counts.RG,
                            S100.Anx.Counts.Tany,
S100.Anx.Counts.MG,
                            S100.Anx.Counts.Endo,
S100.Anx.Counts.Unc)

S100.Anx.CellTypes <- as_tibble(S100.Anx.CellTypes)
```

```
S100.Anx.CellTypes <- rename(S100.Anx.CellTypes,
                             All = "S100.Anx.Counts.All",
                             SCN_Neurons =
"S100.Anx.Counts.Neurons",
                             Extra_SCN_Neurons =
"S100.Anx.Counts.ExNeurons",
                             Astrocytes = "S100.Anx.Counts.Astros",
                             Ependymocytes =
"S100.Anx.Counts.Ependy",
                             Oligodendrocytes =
"S100.Anx.Counts.Oligo",
                             NG2_Cells = "S100.Anx.Counts.NG2",
                             Radial_Glia = "S100.Anx.Counts.RG",
                             Tanycytes = "S100.Anx.Counts.Tany",
                             Microglia = "S100.Anx.Counts.MG",
                             Endothelial_Cells =
"S100.Anx.Counts.Endo",
                             Unclassified = "S100.Anx.Counts.Unc")

S100.Anx.CellTypes$Combos <- S100combs

S100.Anx.CellTypes <- relocate(S100.Anx.CellTypes, Combos, .before =
All)

S100.Anx.CellTypes

S100andAnx.Percent <- (S100.Anx.CellTypes[1,c(3:13)]/2649)*100
S100andAnx.Percent <- tibble(cell_type = col_order, percent_exp_both
= t(S100andAnx.Percent))
S100andAnx.Percent$percent <-
S100andAnx.Percent$percent_exp_both[,1]
S100andAnx.Percent <- S100andAnx.Percent[,c(1,3)]
S100andAnx.Percent$cell_type <-
as_factor(S100andAnx.Percent$cell_type)
S100andAnx.Percent

S100andAnx.Plot <- ggplot(S100andAnx.Percent, aes(x= cell_type, y =
percent)) +
  geom_bar(stat="identity", fill = my_pal)

S100andAnx.Plot


##Differential expression test for Astrocytes, day vs night

Astro.DvN.wilcox <- FindMarkers(All.named,
                                test.use = 'wilcox',
                                ident.1 = "Day",
                                group.by = "times",
                                subset.ident = "Astrocytes",
                                random.seed = 42)



VP_Top <- EnhancedVolcano(Astro.DvN.wilcox,
```

```
                            lab = rownames(Astro.NvD.wilcox),
                            x = "avg_log2FC",
                            y = "p_val",
                            pointSize = 1.5,
                            cutoffLineType = "blank",
                            gridlines.major = F,
                            gridlines.minor = F,
                            axisLabSize = 12,
                            title = "",
                            subtitle = "",
                            caption = "",
                            xlim = c(-10,10),
                            ylim = c(0,250),
                            labSize = 4.5,
                            max.overlaps = 2,
                            col = c(rep("royalblue", 4)),
                            borderWidth = 0.5,
                            vlineWidth = 0.5,
                            hlineWidth = 0.5,
                            colAlpha = 0.2)

VP_Astro_A2A10 <-  EnhancedVolcano(Astro.DvN.wilcox,
                                   lab = rownames(Astro.NvD.wilcox),
                                   x = "avg_log2FC",
                                   y = "p_val",
                                   pointSize = 1.5,
                                   cutoffLineType = "blank",
                                   gridlines.major = F,
                                   gridlines.minor = F,
                                   axisLabSize = 12,
                                   title = "",
                                   subtitle = "",
                                   caption = "",
                                   xlim = c(-10,10),
                                   ylim = c(0,250),
                                   labSize = 4.5,
                                   col = c(rep("royalblue", 4)),
                                   selectLab = c("Anxa2",
"S100a10"),
                                   drawConnectors = T,
                                   typeConnectors = "closed",
                                   colConnectors = "black",
                                   maxoverlapsConnectors = 6,
                                   max.overlaps = 6,
                                   borderWidth = 0.5,
                                   vlineWidth = 0.5,
                                   hlineWidth = 0.5,
                                   colAlpha = 0.2)


ggsave(filename = "/R/Plots/VP_Astro_Top.pdf",
       plot = VP_Top,
       device = "pdf",
       height = 10,
       width = 8,
       units= "in",
```

```
        dpi = 600)

ggsave(filename = "/R/Plots/VP_Astro_A2A10.pdf",
        plot = VP_Astro_A2A10,
        device = "pdf",
        height = 10,
        width = 8,
        units= "in",
        dpi = 600)


##Linear Mixed Model to analyse meta-analysis of acute amplitude and
phase-shift data

AcuteAmp <- as_tibble(read_excel('R/Data/AA_LMM.xlsx'))
AcuteAmp <- rename(AcuteAmp, Amp = 'Acute Amp')
AcuteAmp <- mutate(AcuteAmp,
                     Window = as.factor(Window),
                     Treatment = as.factor(Treatment),
                     SCN = as.factor(SCN))

modAA <- lme(Amp ~ Window*Treatment, data = AcuteAmp, random =
~1|SCN)
anova(modAA)

emm.modAA <- emmeans(modAA, ~ Treatment | Window, adjust = 'sidak')
contrast(emm.modAA, "trt.vs.ctrl")

PhaseShift <- as_tibble(read_excel('/R/Data/PS_LMM.xlsx'))
PhaseShift <- mutate(PhaseShift,
                     Window = as.factor(Window),
                     Treatment = as.factor(Treatment),
                     SCN = as.factor(SCN))

modPS <- lme(Shift ~ Window*Treatment, data = PhaseShift, random =
~1|SCN)
anova(modPS)

emm.modPS <- emmeans(modPS, ~ Treatment | Window, adjust = 'sidak')
contrast(emm.modPS, "trt.vs.ctrl")
```
